# β-actin mediated H3K27ac changes demonstrate the link between compartment switching and enhancer-dependent transcriptional regulation

**DOI:** 10.1186/s13059-023-02853-9

**Published:** 2023-01-25

**Authors:** Syed Raza Mahmood, Nadine Hosny El Said, Kristin C. Gunsalus, Piergiorgio Percipalle

**Affiliations:** 1 Center for Genomics and Systems Biology, New York University Abu Dhabi (NYUAD), P.O. Box 129188, Abu Dhabi, United Arab Emirates; 2 Department of Biology, New York University, New York, NY 10003 USA; 3 Program in Biology, Division of Science and Mathematics, New York University Abu Dhabi (NYUAD), P.O. Box 129188, Abu Dhabi, United Arab Emirates; 4 Department of Biology, Center for Genomics and Systems Biology, New York University, New York, NY 10003 USA; 5 Department of Molecular Biosciences, The Wenner-Gren Institute, Stockholm University, 106 91 Stockholm, Sweden

**Keywords:** 3D genome organization, Enhancer regulation, Transcriptional regulation, Nuclear actin

## Abstract

**Background:**

Recent work has demonstrated that three-dimensional genome organization is directly affected by changes in the levels of nuclear cytoskeletal proteins such as β-actin. The mechanisms which translate changes in 3D genome structure into changes in transcription, however, are not fully understood. Here, we use a comprehensive genomic analysis of cells lacking nuclear β-actin to investigate the mechanistic links between compartment organization, enhancer activity, and gene expression.

**Results:**

Using HiC-Seq, ATAC-Seq, and RNA-Seq, we first demonstrate that transcriptional and chromatin accessibility changes observed upon β-actin loss are highly enriched in compartment-switching regions. Accessibility changes within compartment switching genes, however, are mainly observed in non-promoter regions which potentially represent distal regulatory elements. Our results also show that β-actin loss induces widespread accumulation of the enhancer-specific epigenetic mark H3K27ac. Using the ABC model of enhancer annotation, we then establish that these epigenetic changes have a direct impact on enhancer activity and underlie transcriptional changes observed upon compartment switching. A complementary analysis of fibroblasts undergoing reprogramming into pluripotent stem cells further confirms that this relationship between compartment switching and enhancer-dependent transcriptional change is not specific to β-actin knockout cells but represents a general mechanism linking compartment-level genome organization to gene expression.

**Conclusions:**

We demonstrate that enhancer-dependent transcriptional regulation plays a crucial role in driving gene expression changes observed upon compartment-switching. Our results also reveal a novel function of nuclear β-actin in regulating enhancer function by influencing H3K27 acetylation levels.

**Supplementary Information:**

The online version contains supplementary material available at 10.1186/s13059-023-02853-9.

## Background

Enhancers can communicate with their target promoters over large genomic distances [1]. To facilitate such communication, eukaryotic genomes are highly organized in 3D nuclear space at multiples scales [1]. Rapid advances in sequencing technologies and proximity ligation assays such as HiC have allowed the genome-wide characterization of 3D chromatin interactions [2]. Such studies have revealed that chromosomes are organized into a multi-layered hierarchical structure which plays an essential role in gene regulation [3]. Chromatin folding into insulated loops gives rise to topologically associating domains while interactions among epigenetically similar domains segregate the genome into functionally distinct A and B compartments [3, 4]. Such A/B compartments reflect segregation of the genome into domains that preferentially interact with regions in other A or B compartments and are identified using principal component analysis (PCA) analysis of genome-wide chromatin interaction data [3]. Notably, multiple studies have demonstrated that transcriptional changes during development and differentiation are accompanied by extensive switching of genes between gene-active A and gene-inactive B compartments [5–10]. The precise mechanisms driving changes in gene expression upon compartment reorganization, however, are yet to be fully understood. Similarly, the contribution of different levels of 3D genome architecture to enhancer-promoter communication remains an area of intense debate. Here, using a combination of epigenetic, transcriptional and chromatin interaction data, we show that changes in transcription associated with compartment switching are highly correlated with the activation of distal regulatory elements.

## Results

Changes in 3D genome organization arise naturally over the course of development and differentiation [5–10] or can be triggered by genetic perturbation of proteins involved in genome regulation [11–13]. In order to investigate the mechanisms driving transcriptional change upon compartment-switching in both these scenarios, we utilized two published datasets containing HiC, ATAC-Seq, ChIP-Seq, and RNA-Seq data. The first dataset probes changes in chromatin architecture during reprogramming of mouse embryonic fibroblasts (MEFs) into pluripotent stem cells (PSCs) [14] and allows us to study the relationship between compartment switching and transcription in the context of cell fate change. The second dataset compares the genome architecture of wild-type MEFs with MEFs lacking nuclear β-actin and helps us study changes in transcriptional regulation induced by perturbation of a single regulatory protein known to influence compartment organization [13]. Together, these datasets allow us to study gene regulation within compartment-switching regions in both stably dividing cells and in cells undergoing transcriptional reprogramming.

### Compartment switching regions represent hotspots of transcriptional and epigenetic change

**Fig. 1:**
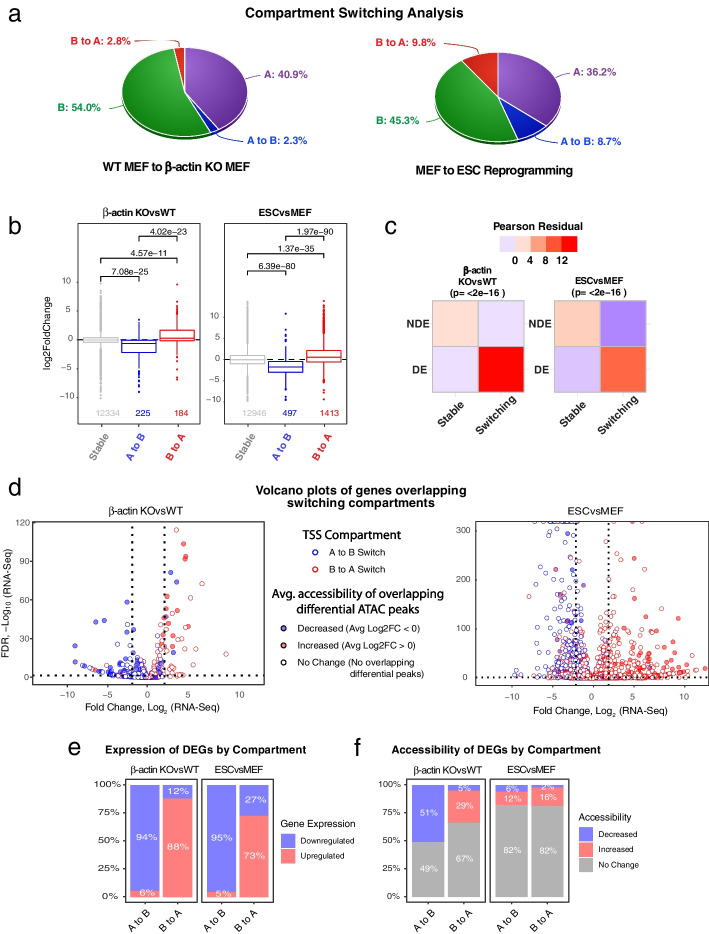
Switching compartments are hotspots of epigenetic and transcriptional changes. **a** Pie charts showing distribution of switching and stable compartments at 50 kb resolution. **b** Boxplots showing average log2FoldChange in RNA-Seq expression for genes overlapping different compartments. Boxes represent first and third quartiles with line in the box showing median and whiskers showing data within 1.5 × interquartile range. *p*-values based on two-tailed, two-sample Wilcoxon-rank sum test. **c** Heatmap showing Pearson residuals and *p*-value based on Pearson’s chi-squared test with Yates’ continuity correction. DEG, differentially expressed genes (absolute log2FC > 2 and padj <  = 0.05), NDE, non-differentially expressed genes. **d** Volcano plots showing expression, accessibility and compartment of all genes overlapping switching (A to B or B to A) regions. *p*-values based on two-tailed Wald test corrected for multiple testing using Benjamini–Hochberg procedure. **e** Bar plots showing percentage of differentially expressed genes up (log2FC > 2 and padj <  = 0.05) or downregulated (log2FC < -2 and padj <  = 0.05) in A to B and B to A switching compartments. **f** Bar plots showing percentage of differentially expressed genes showing increased (log2FC > 0) or decreased (log2FC < 0) average accessibility in A to B and B to A switching compartments

To study the link between 3D genome organization and transcription in the context of cell fate change, we first analyzed compartment switching in MEFs undergoing reprogramming into PSCs. We observed drastic changes in compartment organization with approximately 20% of the genome switching from A to B or B to A state (Fig. 1a). As expected, such compartment switching during MEF to PSC reprogramming was highly correlated with transcriptional changes (Fig. 1b). Since we have recently shown that the loss of nuclear β-actin can have a major impact on the transcriptomic and epigenetic landscape and can drive changes in 3D genome organization [13], we then analyzed compartment switching in β-actin knockout cells. Approximately, 6% of all genomic regions were observed to switch from A to B or B to A compartment upon β-actin loss and this compartment switching was also highly correlated with changes in transcription (Fig. 1a and b). To demonstrate the impact of compartment switching on transcription more clearly, we classified genes overlapping switching and stable compartments as either differentially expressed (DE) or non-differentially expressed (NDE) and performed a chi-square residual analysis. We found that DE genes were significantly overrepresented in compartment-switching regions implying that these regions represented hotspots of transcriptional change (Fig. 1c). Interestingly, while our data confirmed that transcription was highly correlated with compartment level changes, we observed no correspondence between gene expression and changes in topologically associating domains (TADs) (Additional file 1: Fig. S1a and b). Despite showing significant changes in their transcriptional landscape, β-actin knockout cells exhibited negligible changes in TAD structure (Additional file 1: Fig. S1a) suggesting that large-scale transcriptional changes do not always necessitate TAD reorganization. On the other hand, while MEF to PSC reprogramming induced a global weakening of TADs (Additional file 1: Fig. S1a), there was no clear correlation between changes in TAD insulation and direction of transcriptional change (Additional file 1: Fig. S1b). These findings are consistent with several recent studies suggesting that the potential link between transcriptional regulation and TAD insulation maybe highly complex and context dependent [12, 15–18].

Having confirmed that compartment switching regions were enriched for differentially expressed genes, we then asked if these genes also showed significant changes in chromatin accessibility. To answer this question, we leveraged ATAC-Seq data from both datasets. We performed a differential peak calling analysis to identify open chromatin regions showing significant changes in accessibility and calculated the average accessibility of peaks overlapping every compartment switching gene (including its upstream promoter region). We used this measure as a proxy for gene accessibility and integrated it into volcano plots showing expression of compartment switching genes. This analysis allowed us to visualize the relationship between compartment switching, transcription and chromatin accessibility (Fig. 1d).

As expected, this analysis demonstrated that transcription was highly correlated with compartment switching in both datasets. Most genes located in A to B and B to A regions were significantly down and upregulated respectively. Non-switching A and B compartments, on the other hand, showed no specific trend in transcription and contained similar numbers of upregulated and downregulated genes (Additional file 1: Fig. S2). Surprisingly, our results also showed that only a small subset of compartment switching genes exhibited increased or decreased chromatin accessibility. Even with very liberal thresholds for classifying a gene as differentially accessible (absolute ATAC-peak avg.log2FC > 0 and no *p*-value cutoff), majority of the genes in compartment switching regions did not show differential accessibility (Fig. 1d and f). This effect was especially pronounced in B to A switching regions where 73% of differentially expressed genes (DEGs) showed upregulation upon MEF to PSC reprogramming, but only 16% showed increased accessibility (Fig. 1e and f). Similarly, while 88% of DEGs in B to A regions were upregulated upon β-actin loss, only 29% showed increased accessibility (Fig. 1d and f). Our analysis therefore suggested that only a subset of transcriptional changes observed upon compartment switching were dependent on changes in gene accessibility.

To investigate this observation further, we then performed a compartment-wise differential analysis of all ATAC-Seq peaks irrespective of whether they overlapped a gene or not (Additional file 1: Fig. S3a). Contrary to our previous findings based on gene-overlapping peaks, this global analysis showed a clear correspondence between accessibility and compartment switching. Most B to A overlapping peaks showed increased accessibility while most A to B peaks showed decreased accessibility. (Additional file 1: Fig. S3a). Furthermore, a chi-squared residual analysis revealed that just like DEGs, differentially accessible peaks were also enriched in compartment-switching regions (Additional file 1: Fig. S3b). The previously observed limited impact of these changes on gene accessibility, however, was explained by the fact that up to half of all differential ATAC-Seq peaks were located in intergenic regions (Additional file 1: Fig. S3c).

### β-actin loss induces widespread accumulation of enhancer associated histone mark H3K27ac

**Fig. 2:**
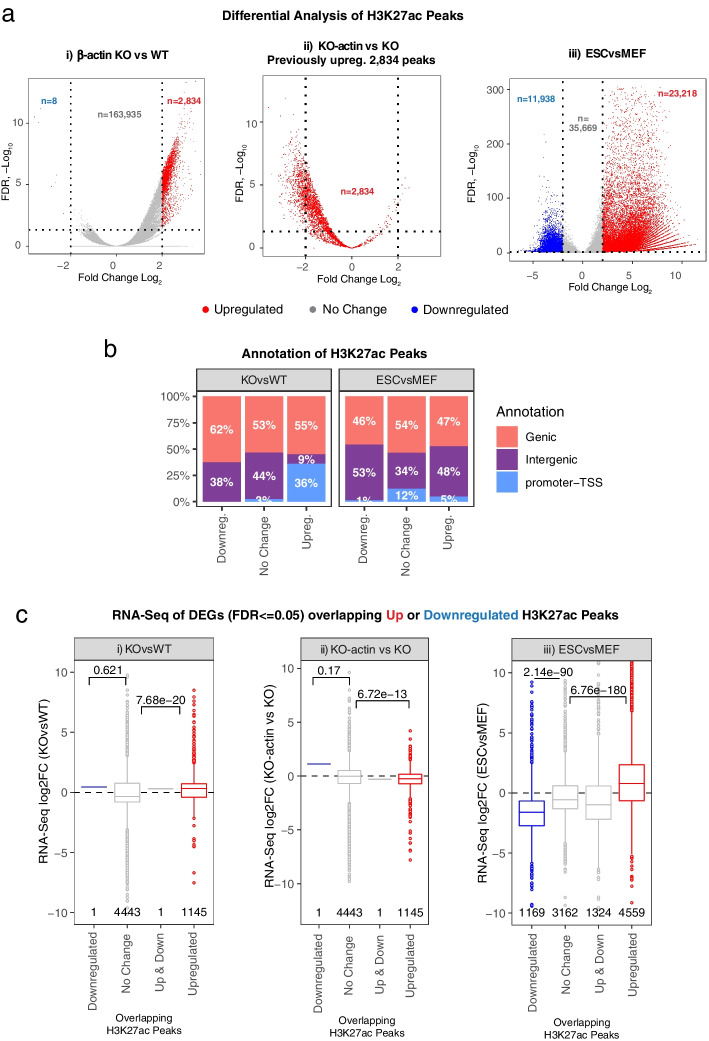
Changes in H3K27ac correlate with changes in genome organization and transcription within compartment switching regions. **a** Volcano plot showing differential analysis of all H3K27ac peaks in (i) WT and β-actin KO cells (ii) β-actin knockout and KO-actin cells iii) MEFs and ESCs. *p*-values based on two-tailed Wald test corrected for multiple testing using Benjamini–Hochberg procedure. Peaks showing log2FC >  = 2 and log2FC <  =  − 2 with padj <  = 0.05 were defined as upregulated and downregulated peaks respectively. **b** Barplots showing annotation of upregulated and downregulated H3K27ac peaks. **c** Boxplots showing average change in RNA-Seq expression for differentially expressed genes (FDR <  = 0.05) overlapping downregulated H3K27ac peaks, unchanging H3K27ac peaks, both upregulated and downregulated H3K27ac peaks and upregulated H3K27ac peaks: (i) average log2FC in KOvsWT expression for genes overlapping WT /KO H3K27ac peaks, (ii) average log2FC in KO-actin vs KO expression for genes overlapping WT/KO H3K27ac peaks, (iii) average log2FC in ESCvsMEF expression for genes overlapping ESC/MEF H3K27ac peaks. Number of genes in each category shown at the bottom. *p*-values based on two-tailed, two-sample Wilcoxon-rank sum test

Since intergenic regions are known to carry distal regulatory elements such as enhancers [19], we hypothesized that accessibility changes overrepresented in compartment switching regions may reflect regulation of enhancer activity. To test the hypothesis, we first investigated if upregulation of genes within B to A switching regions was accompanied by accumulation of enhancer associated epigenetic marks such as H3K27ac. We conducted H3K27ac ChIP-Seq experiments in wild-type and β-actin knockout MEFs and performed a differential analysis of H3K27ac peaks between the two cell types. Our results revealed a striking increase in H3K27ac in β-actin knockout MEFs (Fig. 2a (i)). As we have previously shown that reintroduction of NLS-tagged β-actin into knockout MEFs can reverse B to A compartment switching induced by β-actin loss (*13*) (Additional file 1: Fig. S4a), we then asked if expression of NLS-tagged β-actin could also reverse the observed increase in H3K27ac. To answer this question, we conducted H3K27ac ChIP-Seq in previously generated β-actin knockout cells expressing NLS-tagged β-actin [20] (labeled as KO-actin). Remarkably, differential analysis between KO-actin and KO cell lines confirmed that the peaks upregulated by the loss of β-actin were downregulated upon the reintroduction of NLS-tagged β-actin (Fig. 2a (ii)). This analysis demonstrated a direct link between β-actin levels and H3K27ac.

While it is currently not known how the loss of nuclear β-actin may induce an increase in histone acetylation, previous studies have shown that actin can associate with histone acetyltransferases such as PCAF (p300/CREB binding protein associated factor) [21] and hATAC (histone-modifying complex human Ada-Two-A-containing) [22]. In fact, actin binding to the KAT14 sub-unit of the hATAC complex has been shown to inhibit its acetyl transferase activity [22], a finding consistent with the increased acetylation levels observed in β-actin knockout cells. Similarly, it has also been shown that nuclear actin can regulate histone deacetylase activity [23], another potential pathway via which loss of actin may influence histone acetylation and downstream enhancer function.

Differential analysis of H3K27ac ChIP-Seq upon MEF to PSC reprogramming also revealed a similar increase in H3K27ac (Fig. 2a (iii)). Moreover, annotation of upregulated peaks in both datasets showed that the acetylation increase was largely restricted to genic and intergenic regions and could therefore represent activation of distal regulatory elements like enhancers (Fig. 2b). Only 5% and 36% of upregulated peaks overlapped promoters in MEFvsPSCs and β-actin KOvsWT datasets respectively (Fig. 2b). A compartment-wise analysis of H3K27ac changes revealed that A to B and B to A switching regions showed more pronounced changes in H3K27ac than stable compartments suggesting that the transcriptional changes in these regions were possibly linked to changes in acetylation levels (Additional file 1: Fig. S4b). Similarly, switching regions also showed the most prominent rescue of H3K27ac levels upon the expression of NLS-tagged β-actin (Additional file 1: Fig. S4b).

To more closely study the link between transcription and H3K27ac, we then annotated all differentially expressed genes (FDR <  = 0.05 and no logFC cuttoff) with their closest (in case of intergenic peaks) or overlapping (in case of genic peaks) H3K27ac peaks. We classified each gene as being associated with one or more upregulated peaks, downregulated peaks, both upregulated and downregulated peaks or non-differential peaks and compared their RNA-Seq expression changes. As expected, genes associated with upregulated H3K27ac peaks in β-actin KO cells showed an increase in expression compared to genes associated with non-differential peaks (Fig. 2c (i)). To confirm that these transcriptional and H3K27ac changes were linked to β-actin levels, we then generated RNA-Seq data comparing KO-actin and KO cell lines. Our results confirmed that the reintroduction of NLS-tagged β-actin not only resulted the downregulation of previously upregulated H3K27ac peaks but also resulted in decreased expression of genes associated with these peaks (Fig. 2c (ii)). A complementary analysis of acetylation levels and transcriptional changes in the ESC vs MEF dataset revealed similar results demonstrating the link between transcription and acetylation of non-promoter regions inside compartment switching regions (Fig. 2c (iii)). Together, our findings hinted at a novel role for β-actin levels in regulation of distal regulatory elements and highlighted the relationship between compartment-level organization, H3K27ac and transcription.

### Differential enhancer activity explains transcriptional changes observed upon compartment switching

As our data suggested that compartment switching was potentially associated with changes in enhancer regulation, we then decided to annotate putative enhancers in each of the four cell types under investigation (wild-type MEFs, β-actin knockout MEFs and PSCs). To identify candidate enhancer regions, we utilized the Activity-by-Contact (ABC) model of enhancer identification. This model predicts cell-type specific enhancers using a combination of HiC-Seq, H3K27ac ChIP-Seq and ATAC-Seq data [24] and has been shown to identify distal regulatory elements linked to disease causing genes with a high degree of precision [25]. Briefly, the model proposes that a candidate regulatory element’s ability to influence the expression of a target gene is a function of its “Activity” (measured as the geometric mean of H3K27ac and ATAC-Seq read counts at the element) and “Contact” (measured as the normalized HiC contact frequency between the element and its target promoter) [24]. We utilized cell-type specific ATAC-Seq, H3K27ac ChIP-Seq, and HiC-Seq data to annotate enhancer-promoter interactions in both datasets using the ABC-model.

**Fig. 3:**
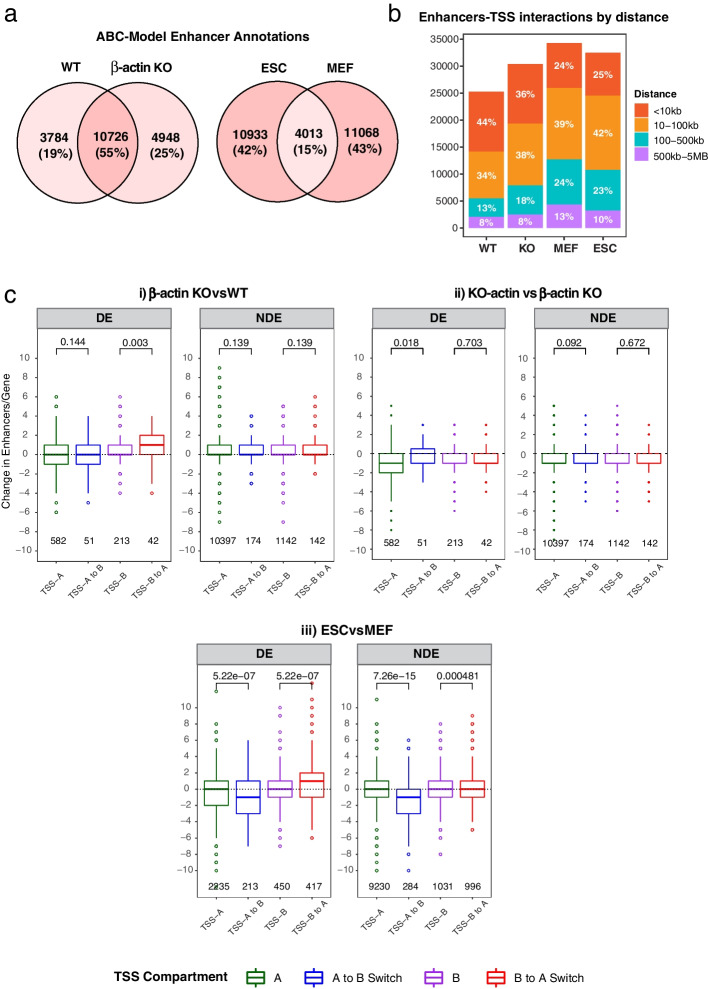
Switching compartments show significant gain or loss of enhancer-promoter contacts. **a** Venn diagrams comparing the number of unique enhancers annotated in WT versus β-actin KO MEFs and MEFs versus ESCs using the ABC model. **b** Bar plot showing the distribution of all enhancer-promoter contacts by distance. **c** Boxplots showing the change in the number of enhancers annotated per gene in different compartments for (i) β-actin KOvsWT, (ii) KO-actin vs β-actin KO, and (iii) ESCvsMEF. Boxes represent first and third quartiles with line in the box showing median and whiskers showing data within 1.5 × interquartile range. *p*-values based on two-tailed, two-sample Wilcoxon-rank sum test. DEG, differentially expressed genes (absolute log2FC > 2 and padj <  = 0.05), NDE, non-differentially expressed genes

To confirm that the ABC model was well-calibrated in each of our cell types, we first analyzed the average number of enhancer predictions per gene. Our results showed that the majority of genes were associated with 1–5 predicted enhancers with an average of two enhancers per gene, a result which was in line with the recommended prediction parameters of the ABC model (Additional file 1: Fig. S5). A comparison of predicted enhancer elements between cell types revealed that wild-type and β-actin knockout cells shared 55% of predicted enhancers with approximately equal proportion of the remaining enhancers being unique to each cell line (Fig. 3a). MEFs and PSCs, on the other hand, revealed a much more drastic reorganization of the regulatory landscape with only 15% of all predicted enhancers shared between the two cell types (Fig. 3a). Our results also showed that the majority of predicted enhancers were located relatively close to their promoters. Around a third of all predicted enhancers were within 100 kb of their target genes and approximately a quarter within 10 kb (Fig. 3b).

We then asked if the previously observed changes in accessibility and H3K27ac at non-promoter regions represented gain or loss of putative enhancers and could potentially explain differential expression of compartment switching genes. To answer this question, we calculated the average number of enhancers predicted by the ABC model for differentially expressed and non-differentially expressed genes before and after compartment switching in each dataset. Our results clearly showed that genes overlapping switching regions exhibited noticeable changes in the number of predicted enhancers unlike genes overlapping stable compartments (Fig. 3c). DE genes switching from B to A compartment, for instance gained approximately one extra enhancer per gene upon β-actin loss (Fig. 3c (i)) or MEF to PSC reprogramming (Fig. 3c (iii)). Remarkably, genes located in B to A switching compartments which exhibited a net increase in enhancers in KO cells lost these enhancers upon the reintroduction of NLS-tagged β-actin (Fig. 3c (ii)) demonstrating the ability of β-actin levels to influence enhancer function. On the other hand, NDE genes located in the same switching compartments also gained or lost some enhancers but showed negligible change in the median number of enhancers per gene. While these results highlight the general link between enhancer activity and transcription (Fig. 3c), they also show that gain or loss of enhancers alone is not sufficient to influence gene expression in the absence of downstream factors such as transcription factor availability and other forms of transcriptional/post-transcriptional gene regulation. This observation is consistent with the concept of activatable and occluded genes which respectively require either chromatin independent mechanisms like transcription factors or chromatin based derepression mechanisms in addition to enhancer activity to be properly activated [26].

**Fig. 4:**
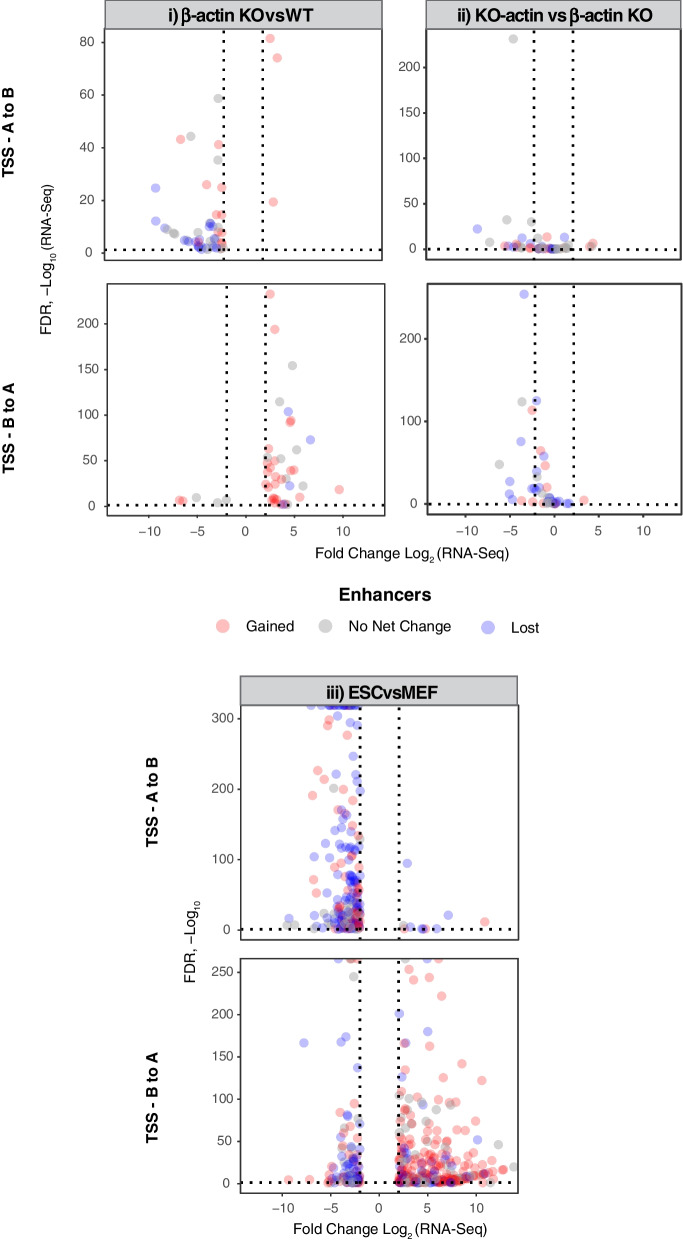
Transcriptional changes in compartment switching regions correlate with gain or loss of enhancers. Volcano plots showing the RNA-Seq expression and FDR of all genes overlapping switching (A to B or B to A) regions for (i) β-actin KOvsWT, (ii) KO-actin vs β-actin KO (note: log2FC and FDR for KO-actin vs KO comparison is shown for genes classified as switching in KOvsWT comparison), and (iii) ESCvsMEF. Red and blue fill represents genes gaining and losing enhancers upon compartment switching while grey fill represents zero net change in enhancers. *p*-values based on two-tailed Wald test corrected for multiple testing using Benjamini–Hochberg procedure

To study if the gain or loss of enhancers annotated using the ABC model correlated with changes in gene expression, we then classified each DE gene as either gaining or losing enhancers and incorporated this information into volcano plots showing the relationship between transcription and compartment switching. In contrast to the previously observed weak association between accessibility of gene bodies and transcription, our results revealed a striking correspondence between gain or loss of predicted enhancers and changes in expression of switching genes (Fig. 4). Furthermore, reintroduction of NLS-tagged β-actin into KO cells was able to reverse transcriptional changes induced by B to A compartment switching confirming the link between actin-dependent H3K27ac changes, compartment organization, and transcription (Fig. 4 (ii)). We then wondered if there was also a relationship between the number of enhancers gained or lost by a gene and the magnitude of its expression change. While we observed a weak linear relationship between these two factors, the number of enhancers gained or lost did not explain a significant proportion of the variation in transcriptional change (Additional file 1: Fig. S6). This finding suggested that enhancer specificity may be more important for determining the magnitude of expression change than the absolute number of enhancer contacts gained or lost by a gene.

### ABC enhancer classification is more strongly influenced by changes in epigenetic state (Activity) than changes in interaction frequencies (Contact)

**Fig. 5:**
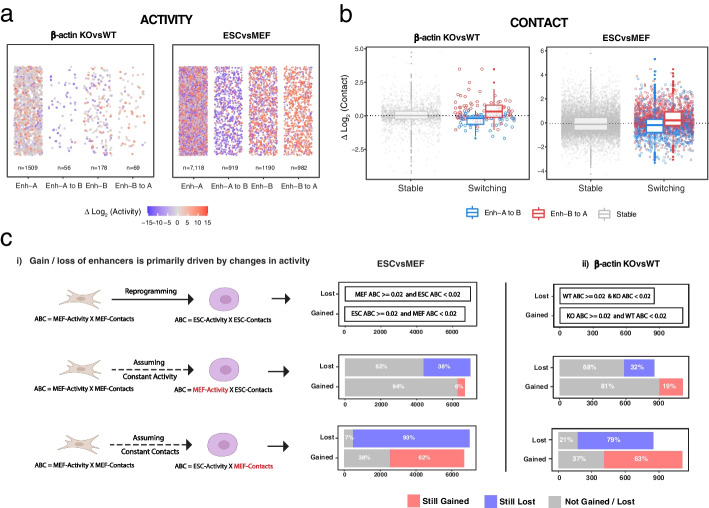
Switching compartments show significant changes in the Activity and Contact frequency of enhancers with their target promoters. **a** Scatterplots showing change in log2(Activity) of unique enhancers between β-actin KO and WT cells and MEFs and ESCs. Only enhancers linked to differentially expressed genes are shown for each compartment. Each dot represents a single unique enhancer. **b** Scatterplots showing change in log2(Contact) of all enhancers with their promoters between β-actin KO and WT cells and MEFs and ESCs. Only enhancers linked to differentially expressed genes are shown for each compartment. Since each enhancer can interact with multiple genes, each dot represents an enhancer-promoter contact rather than a single unique enhancer. **c** (i) Bar plots showing the number of enhancers gained or lost during MEF to ESC reprogramming under assumptions of constant Activity or Contact. Only enhancers linked to differentially expressed genes are shown: (ii) bar plots showing the number of enhancers gained or lost upon β-actin loss under assumptions of constant Activity or Contact

As our results showed that transcriptional change upon compartment-switching could be explained by changes in the enhancer activity, we then decided to investigate the mechanisms driving the activation or deactivation of enhancers in these regions. Since the ABC model annotates enhancers based on a combination of their Activity (chromatin accessibility as measured by ATAC-Seq and acetylation as measured by H3K27ac) and Contact (normalized contact frequency between enhancers and its target promoter), an element can be classified as an enhancer in one cell type and not the other if it exhibits significant changes in either of these components. We therefore asked if epigenetic changes, changes in chromatin interactions or a combination of both, induced the gain or loss of enhancers upon compartment switching. Focusing on enhancers associated with differentially expressed genes, we separately analyzed the change in Activity and Contact of each enhancer element gained or lost upon compartment switching. Our results showed striking changes in the Activity of enhancers located in switching compartments with most B to A switching enhancers gaining Activity and most A to B switching enhancers losing Activity (Fig. 5a). Furthermore, a large proportion of enhancers in switching regions exhibited zero Activity either before or after switching from one compartment to another. For instance, 57% and 62% of all putative enhancers located in A to B regions showed complete loss of Activity after compartment switching in β-actin knockout cells and PSCs respectively (Additional file 1: Fig. S7). The same pattern was observed in B to A regions where a large percentage of gained enhancers had zero Activity before the B to A switch (Additional file 1: Fig. S7). This result suggested that the majority of enhancers in switching regions were completely inactive either before or after compartment switching and hence represented regulatory elements unique to one experimental condition. Similarly, a comparison of Contact frequencies before and after compartment switching showed that A to B switching enhancers, on average, exhibited decreasing contacts with their promoters while B to A switching regions showed increased contacts (Fig. 5b).

While these results showed that changes in both Activity and Contact contributed to the gain or loss of enhancers in switching regions, we wondered if one component was more important than the other in enhancer regulation. To assess the relative contribution of Activity and Contact in enhancer regulation, we therefore adopted a complementary approach. Using the MEF to ESC reprogramming dataset as an example, we first annotated enhancer-promoter contacts using cell-type specific ATAC-Seq, H3K27ac ChIP-Seq and HiC data as before. This analysis showed that 6702 and 7009 DEG linked enhancer-promoter connections were respectively gained and lost during MEF to ESC reprogramming (Fig. 5c (i)). We then asked how these results would change if we assumed that only the Contact frequency of enhancers with their promoters changed during MEF to PSC reprogramming while enhancer Activity remained constant at its original MEF levels. To do this, we annotated ESC enhancers using a combination of MEF Activity (based on MEF-specific ATAC-Seq and H3K27ac ChIP-Seq data) and ESC contacts (based on ESC-specific HiC data). A differential analysis based on these modified ESC enhancer annotations revealed that a large majority of the previously identified differential contacts were not classified as gained or lost under the assumption of constant Activity but were predicted to be shared between the two cell types (Fig. 5c (i)). In contrast, a reciprocal analysis assuming constant Contact and varying Activity still classified 62% and 93% of differential enhancers as being gained or lost. Similar results were obtained using this approach in the β-actin knockout versus wild-type dataset (Fig. 5c (ii)). Together, these results indicated that Activity changes played a much greater role in enhancer classification than changes in chromatin interactions. While this observation is specific to the ABC model and can possibly arise due to technical limitations in detecting low interaction frequencies from HiC data, given the strong correlation observed between ABC enhancer predictions and transcriptional changes (Fig. 4), it may also partly reflect the underlying biology of enhancer regulation. In fact, several studies have suggested that enhancers in pluripotent stem cells are often “poised” by already being in close contact with their target genes [1, 27–29] and hence epigenetic changes rather than changes in chromatin interactions may be more important for their activation. However, due to technical limitations of detecting low frequency chromatin interactions using HiC, the nature of these findings remains preliminary. Future studies utilizing higher resolution contact maps, and cutting-edge techniques such as Capture-C [30] and Micro-C [31] would be essential for probing the interaction landscape of specific enhancers and definitively determining the relative contribution of epigenetic state and interaction frequency in regulating enhancer function.

## Discussion

In this study, we have investigated the mechanisms underlying transcriptional regulation in compartment-switching regions. Our findings reveal that while compartment switching regions act as hotspots for transcriptional change, the majority of genes located in these regions exhibit limited changes in chromatin accessibility. Instead, epigenetic changes at distal regulatory elements play a major role in regulating gene expression within these regions. Using the ABC model of enhancer annotation, we show that B to A switching regions exhibit a net gain of enhancer contacts while A to B switching regions show a net loss. This gain or loss of ABC-based enhancers upon compartment switching is primarily driven by changes in the Activity of these elements (as reflected in ATAC-Seq and H3K27ac ChIP-Seq) and to a lesser extent by changes in chromatin interactions associated with compartment switching. These results demonstrate that the epigenetic and chromatin accessibility changes which drive compartment reorganization are enriched at distal regulatory elements and hence play an important role in both genome organization and transcriptional regulation. Our findings imply that processes like homotypic aggregation and phase separation which are proposed to underlie compartment formation may be more important for regulating enhancer-promoter communication than previously thought. This implication is consistent with several recent studies which demonstrate modest impact of TAD-level changes on gene regulation [17, 32–34] and highlight the importance of homotypic interactions in sculpting the 3D genome [35–37]. Finally, by uncovering a link between β-actin levels, H3K27ac, and enhancer-dependent transcriptional change in compartment switching regions, our work reveals novel roles of nuclear actin in regulating enhancer activity and compartment organization.

## Conclusions

In this study, we have demonstrated that enhancer-dependent transcriptional regulation plays a crucial role in driving gene expression changes associated with compartment-switching. Our results also show that changes in nuclear β-actin levels directly impact H3K27ac and result in dramatic changes in enhancer activity. These findings reveal a novel role of nuclear β-actin in regulating acetylation levels and enhancer function and shed light on the mechanisms that translate changes in 3D genome organization into changes in gene expression.

## Methods

### ChIP-Seq

WT and KO mouse embryonic fibroblasts (MEFs) (from the lab of Dr. Christophe Ampe, University of Gent, Belgium) were maintained and cultured with Dulbecco’s modified Eagle medium (DMEM) with high glucose, 10% fetal bovine serum (FBS), and 100 U/mL penicillin and 100 μg/mL streptomycin, in a humidified incubator with 5% CO_2_ at 37 °C. Cells were crosslinked (two biological replicates per condition and input controls) using 1% formaldehyde (Sigma Cat. No. F8775) for 10 min followed by quenching with 0.125 M Glycine for 5 min and lysis with lysis buffer 1-LB1-(50 mM Hepes KOH pH 7.5, 10 mM NaCl, 1 mM EDTA, 10% glycerol, 0.5% NP-40, 0.25% Triton X-100). Nuclei were pelleted, collected, and washed using lysis buffer 2-LB2- (10 mM Tris–HCl pH 8, 200 mM NaCl, 1 mM EDTA, 0.5 mM EGTA). This was followed by lysis using lysis buffer 3 LB3 (10 mM Tris–HCl pH 8; 100 mM NaCl, 1 mM EDTA; 0.5 mM EGTA; 0.1% Na-Deoxycholate, 0.5% N-laurylsarcosine). Chromatin was sheared using Qsonica Sonicator (4 cycles of 3 min at 70% Amplitude), and then checked on 0.8% agarose gel. One hundred micrograms of fragmented chromatin was mixed with 5 μg of H3K27ac-Anti-Histone H3 (acetyl K27) antibody-ChIP Grade (ab4729) (abcam). The protein-antibody immunocomplexes were recovered by the Pierce Protein A/G Magnetic Beads (Thermo-Scientific). Beads and attached immunocomplexes were washed twice using low salt wash buffer (LS) (0.1% SDS; 2 mM EDTA, 1% Triton X-100, 20 mM Tris–HCl pH 8, 150 mM NaCl) and high salt (HS) wash buffer (0.1% SDS, 2 mM EDTA, 1% Triton X-100, 20 mM Tris–HCl pH 8, 500 mM NaCl), respectively. The beads were resuspended in elution buffer (50 mM Tris–HCl pH 8, 10 mM EDTA, 1% SDS). De-crosslinking was achieved by adding 8 μL 5 M NaCl and incubating at 65 °C overnight. RNase A (1 μL 10 mg/mL) was added for a 30 min incubation at 37 °C. Then, 4 μL 0.5 M EDTA, 8 μL 1 M Tris–HCl, and 1 μL 20 mg/mL proteinase K (0.2 mg/mL) were added for a 2-h incubation at 42 °C to digest the chromatin. DNA was purified by QIAquick PCR purification kit (Qiagen, Germantown, MD, USA) for qPCR analysis and sequencing. Library preparation and sequencing was performed by Novogene Co., Ltd, using standard illumina protocols.

### HiC analysis

Raw HiC data for WT/β-actin-KO MEFs and MEF to PSC reprogramming was downloaded from GSE149986 (Samples GSM4519272-75) and GSE96553 (Samples GSM3714983-86) respectively. Raw sequencing data was processed with the HiCUP pipeline using default settings followed by analysis using HOMER. Processed bam files produced by HiCUP were converted to HOMER format using the script hicup2homer and to homer tag directories using the command makeTagDirectory -format HiCsummary. For both datasets, PCA analysis was performed using HOMER with the command runHiCpca.pl -genome mm10 -res 50,000 -window 50,000 followed by annotation and differential analysis with the scripts annotatePeaks.pl and getDiffExpression.pl. Bins changing PC1 values from positive to negative or vice versa with an FDR of less than 0.05 between conditions were classified as switching. TADs were called on replicate-merged tag directories of the control condition in each dataset (WT-MEFs / Bα cells) using the HOMER function findTADsAndLoops.pl with -window and -res set to 50,000. The insulation score of the identified domains in all replicates of each condition was extracted using the command findTADsAndLoops.pl with the “score” option followed by differential analysis using getDiffExpression.pl. Domains showing absolute change in insulation score of > 0.25 with adjusted *p*-value < 0.05 were classified as differential.

### RNA-Seq analysis

Raw counts for β-actin KO and MEF to PSC reprogramming were downloaded from GSE95830 and GSE129495 respectively. DESeq2 was used to perform differential expression analysis using default settings.

### ATAC-Seq analysis

Raw ATAC-Seq fastq files for β-actin and MEF to PSC reprogramming were downloaded from GSE133196 and GSE113428 respectively. Adapter trimming was performed using trim-galore with default settings. Surviving paired reads were aligned against the relevant reference genome (GRCm38) using Burrows-Wheeler Aligner BWA-MEM. Resulting BAM alignments were cleaned, sorted, and deduplicated (PCR and Optical duplicates) with PICARD tools (http://broadinstitute.github.io/picard). Processed bam files were converted to HOMER tag directories followed by annotation and differential analysis with the scripts annotatePeaks.pl and getDiffExpression.pl. ATAC-Seq peaks were called on cleaned, deduplicated bam files of both replicates of each condition together using macs2 with the parameters -q 0.05 -g mm/hg –keep-dup all –nomodel –shift − 100 –extsize 200 -B –broad -f BAMPE. Peaks of the two conditions being compared were merged using homer command mergePeaks. Differential peaks were identified and annotated using homer scripts annotatePeaks.pl and getDiffExpression.pl. To classify a gene as showing differential accessibility in Fig. 1d, all merged peaks were intersected with genes and their promoter regions (750 bp upstream of TSS) and log2FC for all peaks overlapping a gene was averaged. Genes showing average log2FC > 0.05 were classified as differential.

### ChIP-Seq analysis

Raw ChIP-Seq data for H3K27ac in WT/KO β-actin MEFs was generated as described in the relevant methods section. Raw fastq files for H3K27ac ChIP-Seq in MEF to PSC reprogramming were downloaded from GSE113429. For both datasets, adapter trimming was performed using trim-galore with default settings. Surviving reads were aligned against the relevant reference genome (GRCm38) using Burrows-Wheeler Aligner BWA-MEM. Resulting BAM alignments were cleaned, sorted, and deduplicated (PCR and Optical duplicates) with PICARD tools. For WT/KO β-actin MEFs peaks were called on cleaned, deduplicated bam files of both replicates of each condition together using macs2 with the parameters -q 0.05 -g mm –keep-dup all –broad -f BAMPE. Peaks of the two conditions being compared were merged using homer command mergePeaks. Differential peaks were identified and annotated using homer scripts annotatePeaks.pl and getDiffExpression.pl.

### ABC-Model analysis

ABC model analysis was performed using recommended settings unless specified otherwise (https://github.com/broadinstitute/ABC-Enhancer-Gene-Prediction). To generate a list of candidate promoters, representative transcripts for protein-coding genes were downloaded from the RefSeq Select dataset and 500 bp regions centered on the TSS were regarded as a promoter. To identify candidate enhancer regions, peaks were called on previously generated ATAC-Seq bam files with macs2 using the parameters -p 0.1 –call-summits. The peak file was used with the script makeCandidateRegions.py with the parameters –peakExtendFromSummit 250 –nStrongestPeaks 150,000. Candidate enhancers were separately identified in the control and treatment conditions, merged into a single list using bedtools merge command and resized to 500 bp (where needed). This merged enhancer list was used for downstream analysis. Enhancer activity was quantified with the script run.neighborhoods.py and quantile normalized to the EnhancersQNormRef.K562.txt file using the option -qnorm. To prepare an RNA-Seq expression table used in the script run.neighborhoods.py, RNA-Seq differential analysis was performed using DESEQ2 with default settings and expression of genes for which DESEQ2 analysis computed a *p*-value was set to 1 while expression of other genes was set to zero. To convert HiC data into a format usable by the script compute_powerlaw_fit_from_hic.py, homer tag directories were converted to hic files using the homer script tagDir2hicFile.pl followed by the command juicebox_dump.py. ABC scores were computed using the script predict.py using the options –hic-resolution 5000 –scale_hic_using_powerlaw –threshold 0.02 –make_all_putative. Interactions from the output file EnhancerPredictionsFull.txt were used for downstream analysis. ABC and Contact scores for these interactions in the opposing condition were extracted from the output file EnhancerPredictionsAllPutative.txt for Fig. 3a and 3b. Figure 2c was generated using data from the output file GenePredictionStats.txt.

### Plots and statistical tests

Plots including statistical test results were generated using R packages ggplot2, ggpubr, rstatix, ggcorplot, and ggvenndiagram. R packages tidyverse, valr and broom were used for miscellaneous data processing before plotting.

## Supplementary Information

**Additional file 1:Fig S1.** TAD insulation changes show no correlation with transcription. a) Insulation scores of TADs identified at 50 kb resolution in control condition for each experiment. Insulation scores for the control condition are shown on the x axis and insulation scores for the same domain in the treatment condi- tion are shown on the y axis b) Boxplots showing average log2FoldChange in RNA-Seq expression for genes overlapping differential TADs. Boxes represent first and third quartiles with line in the box showing median and whiskers showing data within 1.5× interquartile range. p-values based on two-tailed, two-sample Wilcoxon-rank sum test. **Fig S2.** Non-switching compartments contain both up and down regulated genes. a) Volcano plots showing expression, accessibility and compartment of all genes overlapping stable A to B compartments. p-values based on two-tailed Wald test corrected for multiple testing using Benjamini–Hochberg procedure b) Bar plots showing percentage of differentially expressed genes up (log2FC>2 & padj<=0.05) or downregulated (log2FC<-2 & padj<=0.05) in A and B compartments. **Fig S3.** Compartment switching correlates with changes in chromatin accessibility. a) Volcano plots showing log2FoldChange in accessibility of all ATAC-Seq peaks overlapping stable and switching compartments. p-values based on two-tailed Wald test corrected for multiple testing using Benjamini– Hochberg procedure b) Heatmap showing pearson residuals and pvalue based on Pearson's. Chi-squared test with Yates' continuity correction. DP=Differential Peak (absolute log2FC>=0.5 & p.adj <=0.05), NDP=Non-Differential Peak (absolute log2FC<0.5 or p.adj >0.05) c) Bar plots showing percentage of genic and intergenic differential peaks in switching compartments. **Fig S4.** Loss of β-actin triggers accumulation of H3K27ac in B to A switching regions. a) β-actin KO over WT Log2FC in rlog/VST normalized H3K27ac counts for each 50kb bin in switching and stable compartments. FIG S5. ABC enhancer predictions lie within recommended parameters in all cell types. Violin plots showing the distribution of average number of enhancers predicted per gene for all datasets. **Fig S6.** Magnitude of transcriptional change shows limited correlation with number of enhancers gained or lost. Scatierplot showing log2FC in RNA-Seq expression on the y-axis and number of enhancers gained or lost on the x-axis for compartment switching genes. **Fig S7.** Majority of enhancers in switching regions show zero Activity before or atier switching. Barplots showing the percentage of enhancers showing zero or non-zero Activity in each compartment. Only gained or lost enhancers linked to differentially expressed genes are shown. **Fig S8.** Gain or loss of enhancers is primarily driven by changes in Activity. Bar plots showing the number of enhancers gained or lost between WT and β-actin KO cells under assumptions of constant Activity or Contact. Only enhancers linked to differentially expressed genes are shown.

**Additional file 2:Table S1.** β-actin WTvsKO RNA-Seq and Enhancer Annotation Data. **Table S2.** MEFvsESC RNA-Seq and Enhancer Annotation Data. **Table S3.** β-actin WTvsKO Differential Analysis of ATAC peaks. **Table S4.** MEFvsESC Differential Analysis of ATAC peaks. **Table S5.** β-actin WTvsKOvsKO-actin Differential Analysis of H3K27ac peaks. **Table S6.** MEFvsESC Differential Analysis of H3K27ac peaks.

**Additional file 3.** 

## Data Availability

Sequencing data for H3K27ac ChIP-Seq in β-actin WT, KO, and KO-actin MEFs has been deposited in the Gene Expression Omnibus under accession numbers GSE196089 and GSE213432. Sequencing data for ATAC-Seq in KO-actin MEFs has been deposited in the Gene Expression Omnibus under accession number GSE213431. Previously published datasets used in this study include: S. R. Mahmood, X. Xie, N. Hosny El Said, T. Venit, K. C. Gunsalus, P. Percipalle, β-actin dependent chromatin remodeling mediates compartment level changes in 3D genome architecture. *Nat Commun*. 12, 5240 (2021). GSE149986 and GSE133196. D. C. Di Giammartino, A. Kloetgen, A. Polyzos, Y. Liu, D. Kim, D. Murphy, A. Abuhashem, P. Cavaliere, B. Aronson, V. Shah, N. Dephoure, M. Stadtfeld, A. Tsirigos, E. Apostolou, KLF4 is involved in the organization and regulation of pluripotency-associated three-dimensional enhancer networks. *Nat Cell Biol*. 21, 1179–1190 (2019). GSE113431. X. Xie, B. Almuzzaini, N. Drou, S. Kremb, A. Yousif, A.-K. Ö. Farrants, K. Gunsalus, P. Percipalle, β-Actin-dependent global chromatin organization and gene expression programs control cellular identity. *FASEB J*. 32, 1296–1314 (2018). GSE95830.
