# Supplementary material for: β-actin mediated H3K27ac changes demonstrate the link between compartment switching and enhancer-dependent transcriptional regulation
Source: Genome Biol. 2023 Jan 25;24:18. doi: 10.1186/s13059-023-02853-9 (PMC9875490; doi:10.1186/s13059-023-02853-9)

SUPPLEMENTARY FIGURES

FIG S1. TAD insulation changes show no correlation with transcription. a) Insulation scores of TADs identified at 50 kb resolution in control condition for each experiment. Insulation scores for the control condition are shown on the x axis and insulation scores for the same domain in the treatment condition are shown on the y axis **b)** Boxplots showing average log2FoldChange in RNA-Seq expression for genes overlapping differential TADs. Boxes represent first and third quartiles with line in the box showing median and whiskers showing data within 1.5× interquartile range. p-values based on two-tailed, two-sample Wilcoxon-rank sum test

FIG S2. Non-switching compartments contain both up and down regulated genes. a) Volcano plots showing expression, accessibility and compartment of all genes overlapping stable A to B compartments. p-values based on two-tailed Wald test corrected for multiple testing using Benjamini–Hochberg procedure **b)** Bar plots showing percentage of differentially expressed genes up (log2FC>2 & padj<=0.05) or downregulated (log2FC<-2 & padj<=0.05) in A and B compartments

FIG S3. Compartment switching correlates with changes in chromatin accessibility. a) Volcano plots showing log2FoldChange in accessibility of all ATAC-Seq peaks overlapping stable and switching compartments. p-values based on two-tailed Wald test corrected for multiple testing using Benjamini–Hochberg procedure **b)** Heatmap showing pearson residuals and pvalue based on Pearson's Chi-squared test with Yates' continuity correction. DP=Differential Peak (absolute log2FC>=0.5 & p.adj <=0.05), NDP=Non-Differential Peak (absolute log2FC<0.5 or p.adj >0.05) **c)** Bar plots showing percentage of genic and intergenic differential peaks in switching compartments

FIG S4. Loss of β-actin triggers accumulation of H3K27ac in B to A switching regions. a) β-actin KO over WT Log2FC in rlog/VST normalized H3K27ac counts for each 50kb bin in switching and stable compartments

FIG S5. ABC enhancer predictions lie within recommended parameters in all cell types. Violin plots showing the distribution of average number of enhancers predicted per gene for all datasets.

FIG S6. Magnitude of transcriptional change shows limited correlation with number of enhancers gained or lost. Scatterplot showing log2FC in RNA-Seq expression on the y-axis and number of enhancers gained or lost on the x-axis for compartment switching genes.

FIG S7. Majority of enhancers in switching regions show zero Activity before or after switching. Barplots showing the percentage of enhancers showing zero or non-zero Activity in each compartment. Only gained or lost enhancers linked to differentially expressed genes are shown.

FIG S8. Gain or loss of enhancers is primarily driven by changes in Activity. Bar plots showing the number of enhancers gained or lost between WT and β-actin KO cells under assumptions of constant Activity or Contact. Only enhancers linked to differentially expressed genes are shown.

Fig S1

a

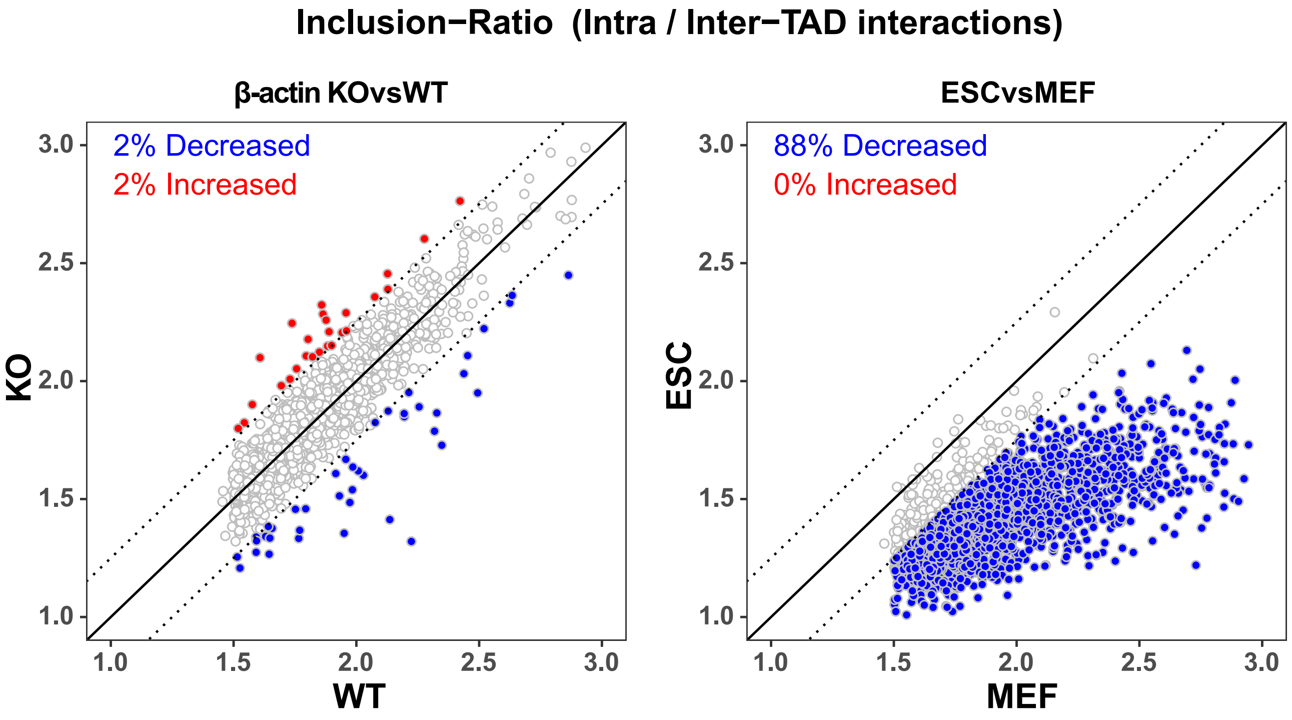

Inclusion−Ratio (Intra / Inter−TAD interactions)
β-actin KOvsWT
2% Decreased
2% Increased
KO
WT
ESCvsMEF
88% Decreased
0% Increased
ESC
MEF
1.0
1.5
2.0
2.5
3.0

b

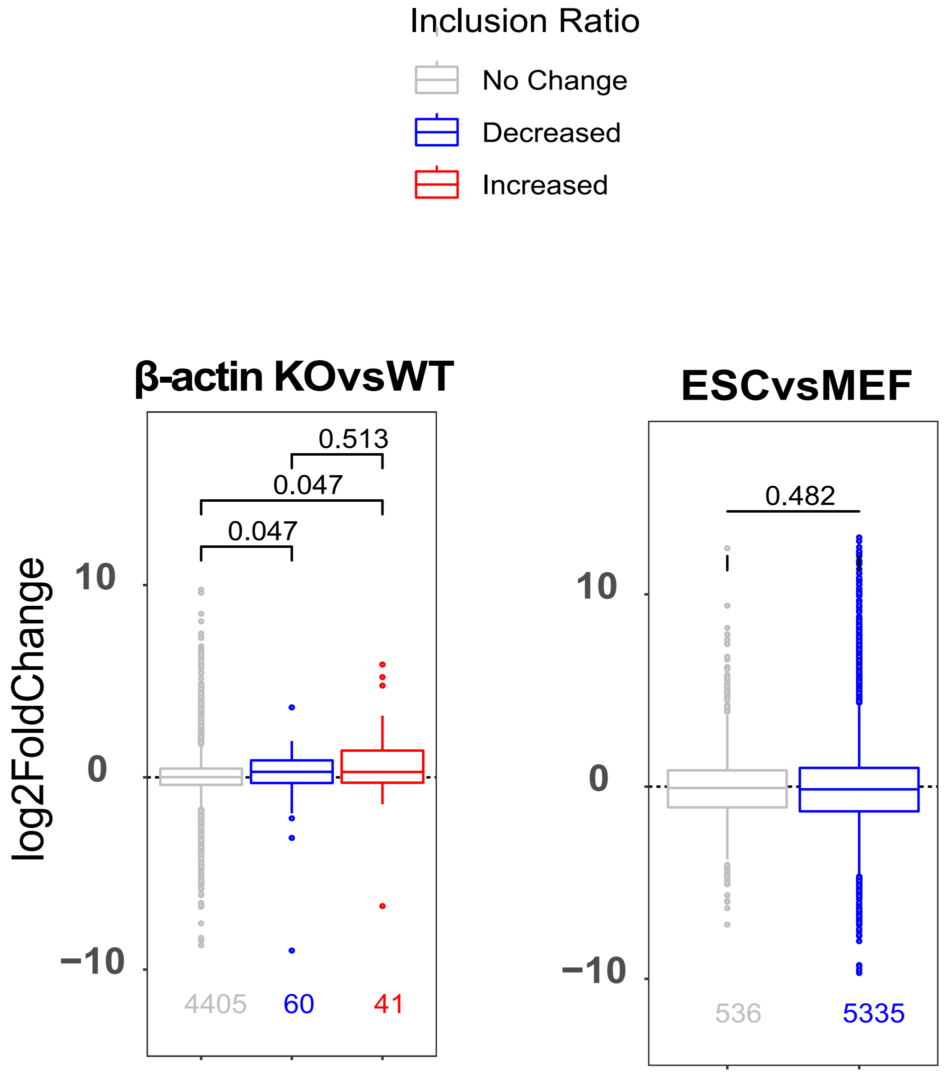

Inclusion Ratio
No Change
Decreased
Increased
β-actin KOvsWT
0.513
0.047
0.047
log2FoldChange
10
0
−10
4405
60
41
ESCvsMEF
0.482
536
5335

Volcano plots of genes overlapping stable compartments

Fig S2

a

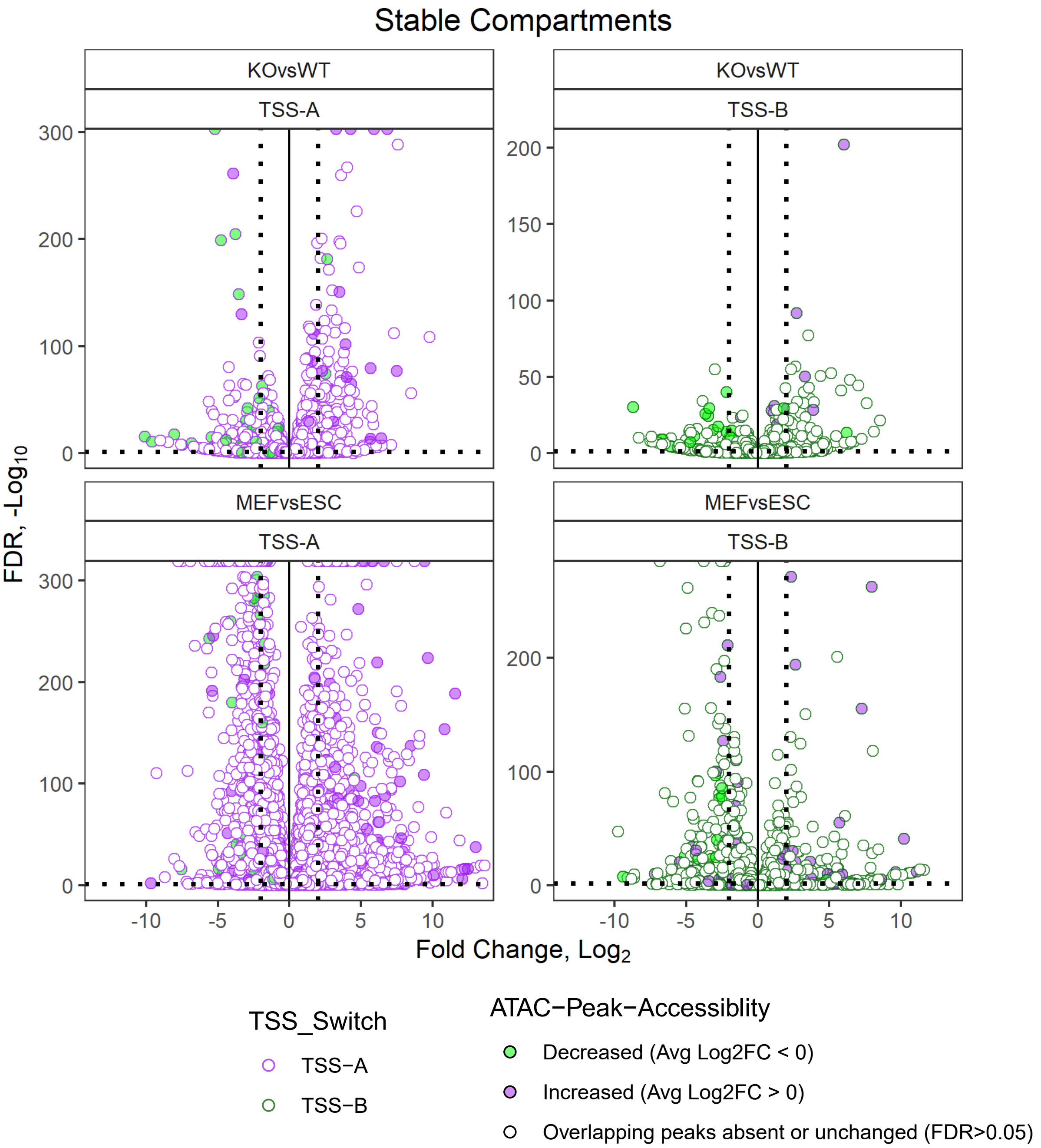

b

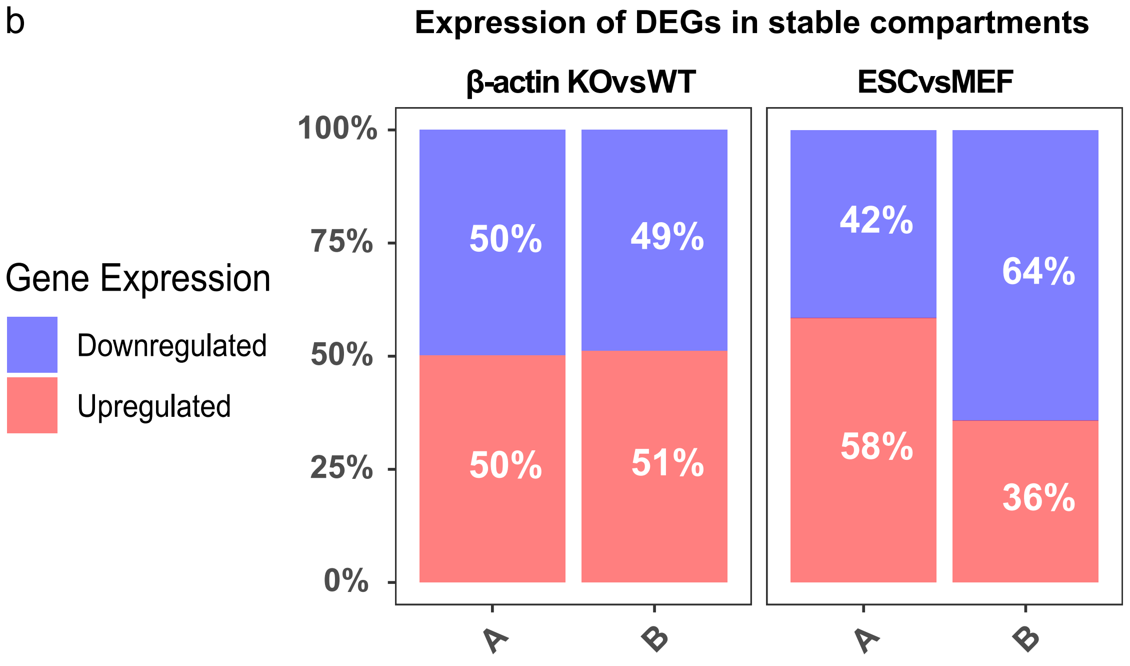

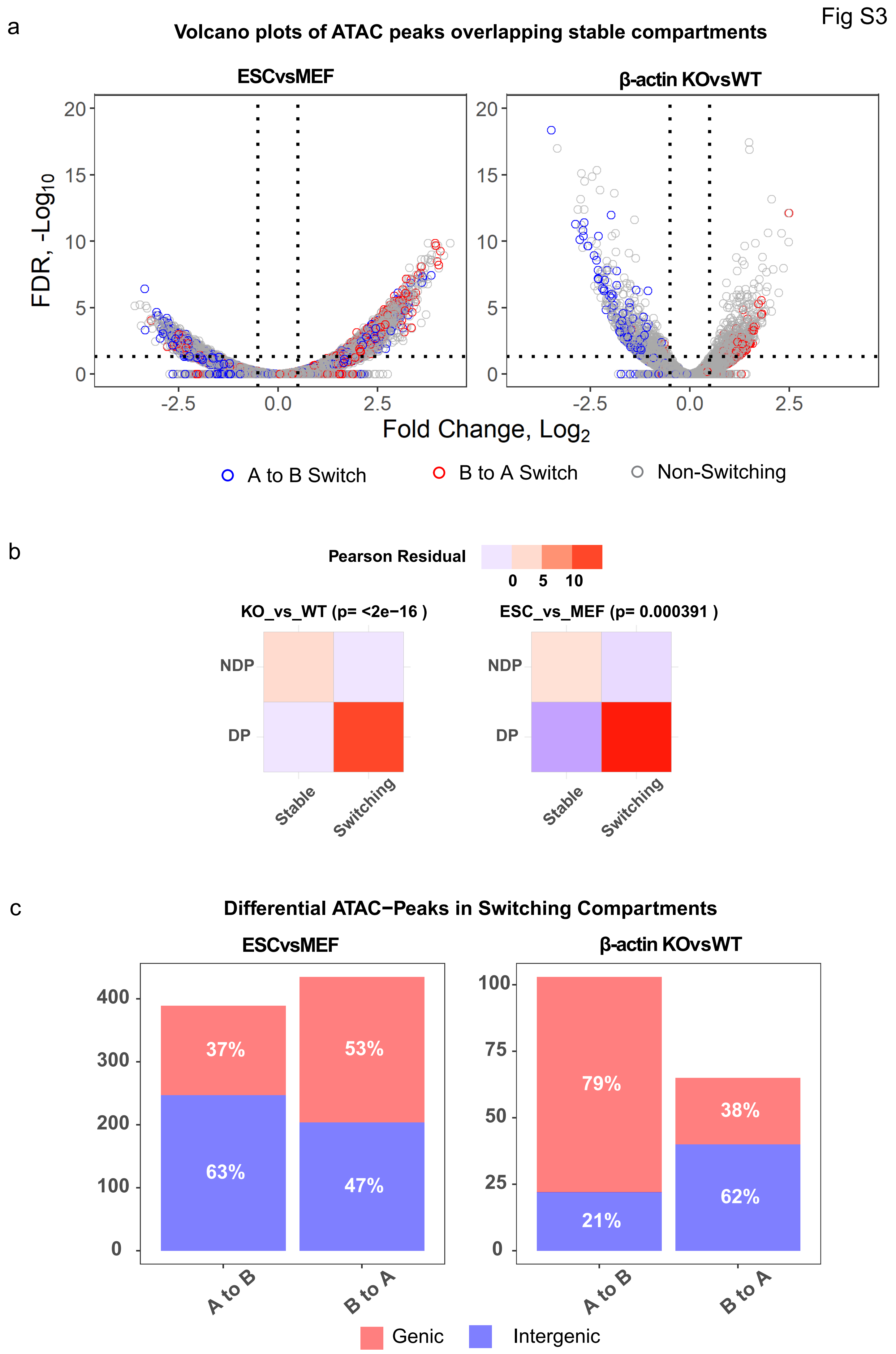
Fig S3
a
Volcano plots of ATAC peaks overlapping stable compartments
ESCvsMEF
β-actin KOvsWT
FDR, -Log10
Fold Change, Log2
A to B Switch
B to A Switch
Non-Switching
b
Pearson Residual
0
5
10
KO_vs_WT (p= <2e−16)
ESC_vs_MEF (p= 0.000391)
NDP
DP
Stable
Switching
c
Differential ATAC−Peaks in Switching Compartments
ESCvsMEF
β-actin KOvsWT
37%
63%
53%
47%
79%
21%
38%
62%
A to B
B to A
Genic
Intergenic

Fig S4

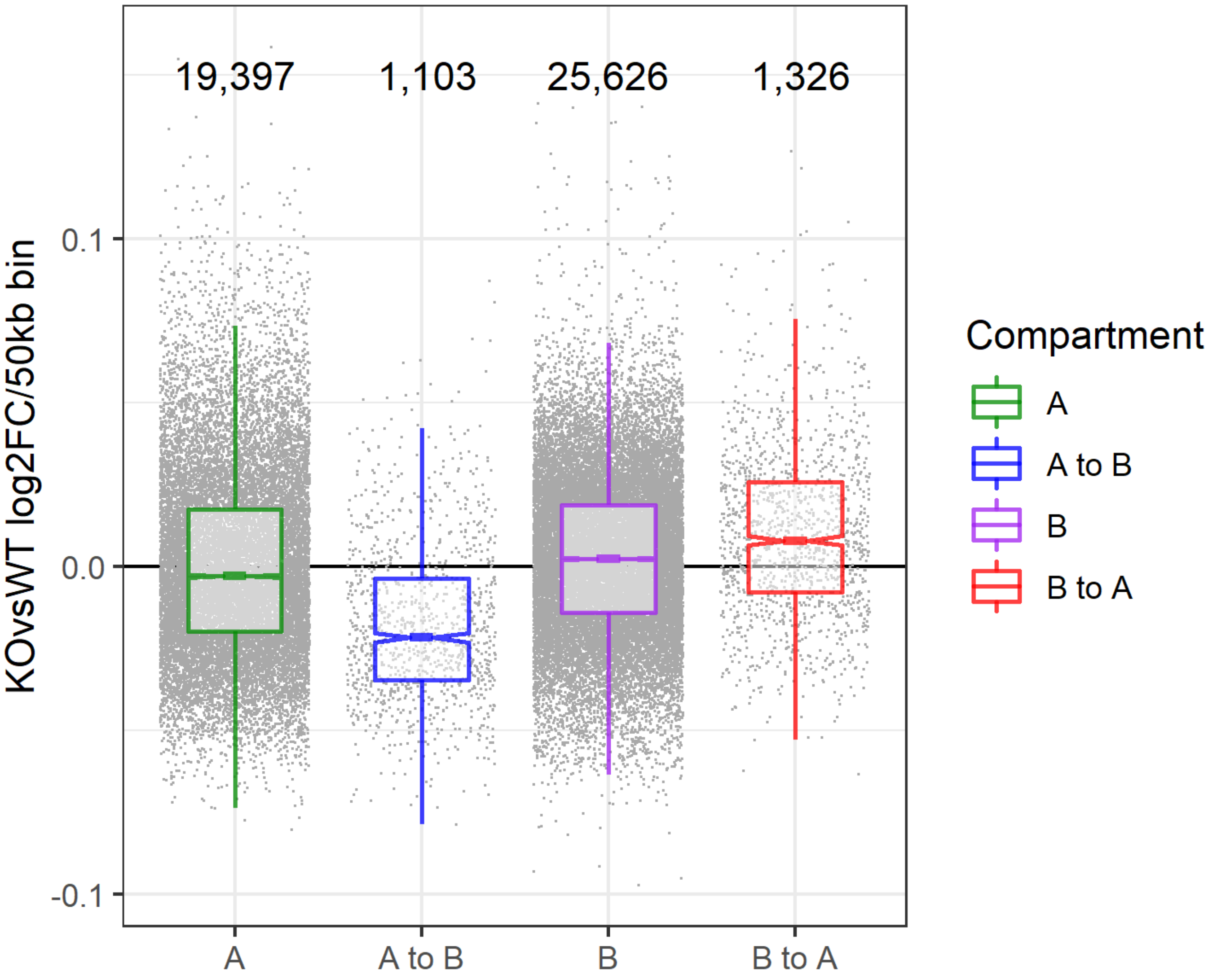
19,397
1,103
25,626
1,326
KOvsWT log2FC/50kb bin
0.1
0.0
-0.1
A
A to B
B
B to A
Compartment
A
A to B
B
B to A

Fig S5

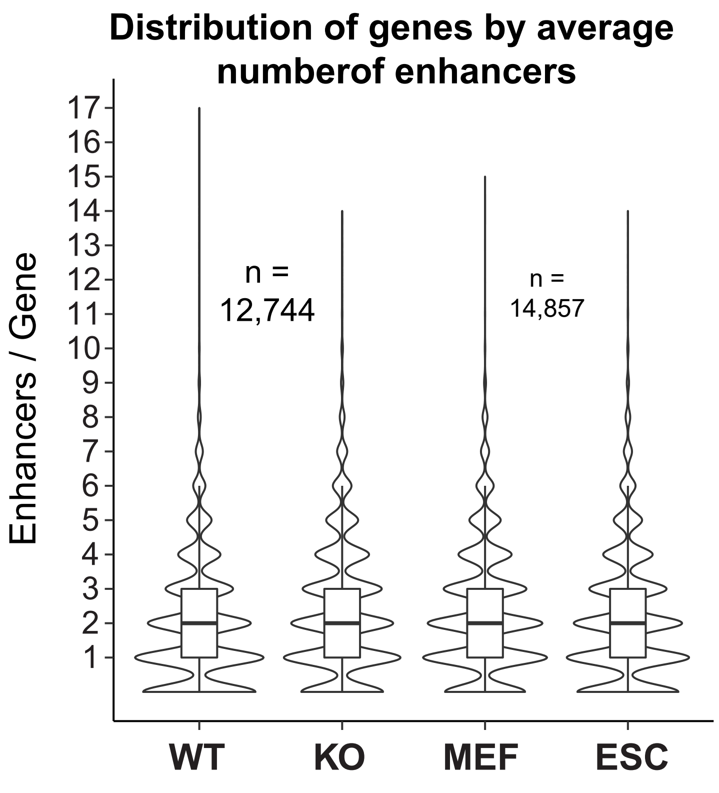
Distribution of genes by average numberof enhancers
Enhancers / Gene
17
16
15
14
13
12
11
10
9
8
7
6
5
4
3
2
1
n = 12,744
n = 14,857
WT
KO
MEF
ESC

Fig S6

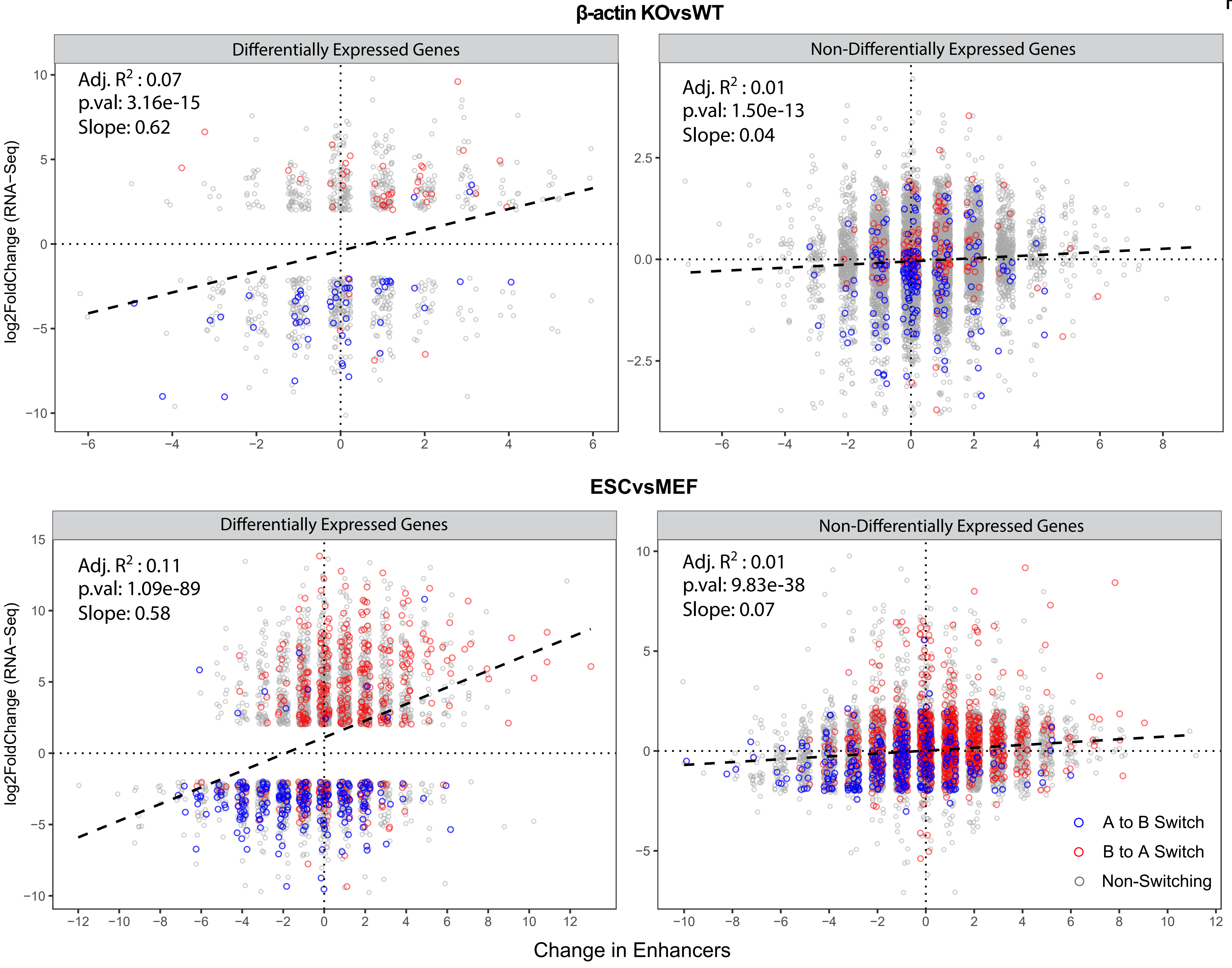
β-actin KOvsWT
Differentially Expressed Genes
Adj. R2 : 0.07
p.val: 3.16e-15
Slope: 0.62
Non-Differentially Expressed Genes
Adj. R2 : 0.01
p.val: 1.50e-13
Slope: 0.04
ESCvsMEF
Differentially Expressed Genes
Adj. R2 : 0.11
p.val: 1.09e-89
Slope: 0.58
Non-Differentially Expressed Genes
Adj. R2 : 0.01
p.val: 9.83e-38
Slope: 0.07
log2FoldChange (RNA-Seq)
Change in Enhancers
A to B Switch
B to A Switch
Non-Switching

Fig S7

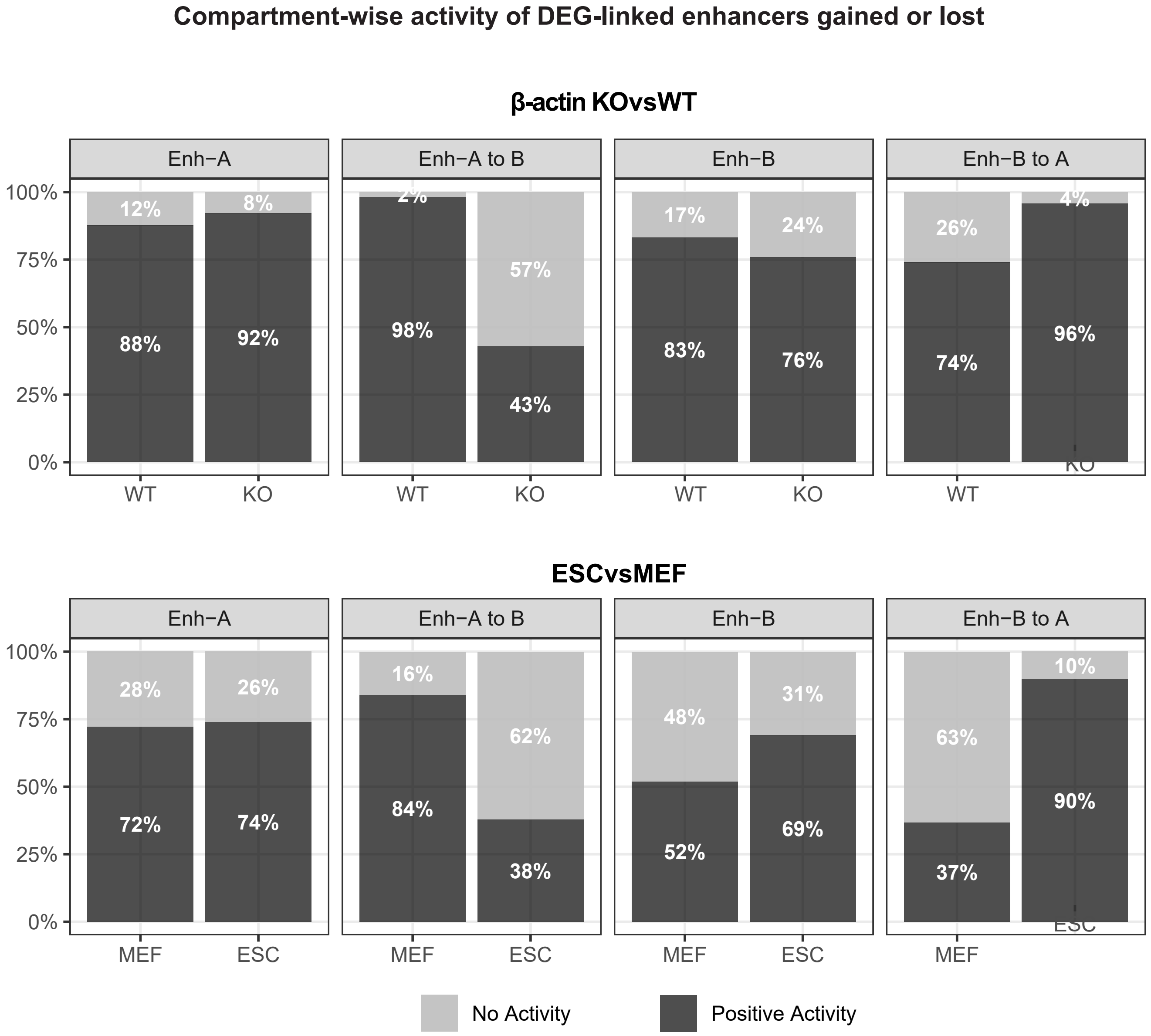
Compartment-wise activity of DEG-linked enhancers gained or lost
β-actin KOvsWT
Enh−A
Enh−A to B
Enh−B
Enh−B to A
100%
75%
50%
25%
0%
12%
8%
88%
92%
2%
98%
57%
43%
17%
24%
83%
76%
26%
4%
74%
96%
WT
KO
ESCvsMEF
28%
26%
72%
74%
16%
84%
62%
38%
48%
31%
52%
69%
63%
10%
37%
90%
MEF
ESC
No Activity
Positive Activity

Changes in activity contribute more to enhancer gain/loss than changes in contact

β-actin KOvsWT

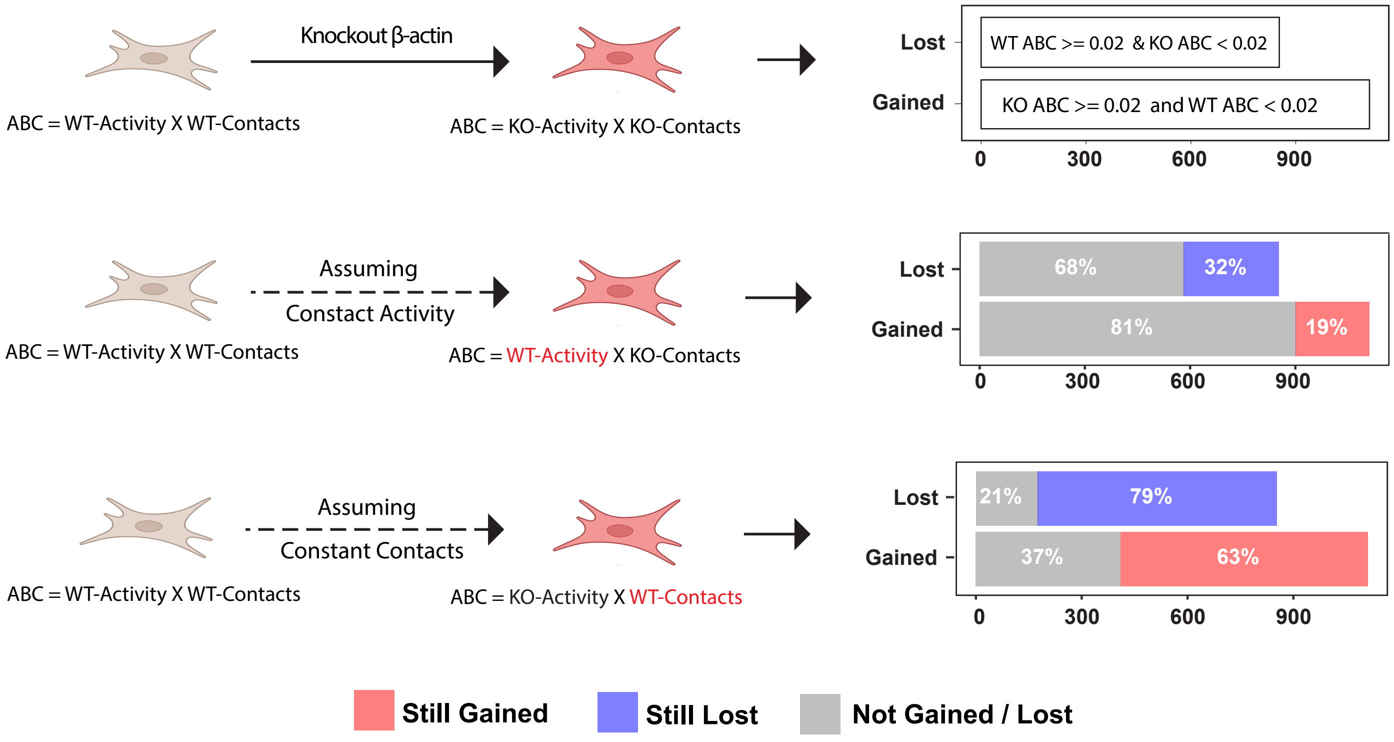

Supplement: Supplementary file 1 — Additional file 1:Fig S1. TAD insulation changes show no correlation with transcription. a) Insulation scores of TADs identified at 50 kb resolution in control condition for each experiment. Insulation scores for the control condition are shown on the x axis and insulation scores for the same domain in the treatment condi- tion are shown on the y axis b) Boxplots showing average log2FoldChange in RNA-Seq expression for genes overlapping differential TADs. Boxes represent first and third quartiles with line in the box showing median and whiskers showing data within 1.5× interquartile range. p-values based on two-tailed, two-sample Wilcoxon-rank sum test. Fig S2. Non-switching compartments contain both up and down regulated genes. a) Volcano plots showing expression, accessibility and compartment of all genes overlapping stable A to B compartments. p-values based on two-tailed Wald test corrected for multiple testing using Benjamini–Hochberg procedure b) Bar plots showing percentage of differentially expressed genes up (log2FC>2 & padj<=0.05) or downregulated (log2FC<-2 & padj<=0.05) in A and B compartments. Fig S3. Compartment switching correlates with changes in chromatin accessibility. a) Volcano plots showing log2FoldChange in accessibility of all ATAC-Seq peaks overlapping stable and switching compartments. p-values based on two-tailed Wald test corrected for multiple testing using Benjamini– Hochberg procedure b) Heatmap showing pearson residuals and pvalue based on Pearson's. Chi-squared test with Yates' continuity correction. DP=Differential Peak (absolute log2FC>=0.5 & p.adj <=0.05), NDP=Non-Differential Peak (absolute log2FC<0.5 or p.adj >0.05) c) Bar plots showing percentage of genic and intergenic differential peaks in switching compartments. Fig S4. Loss of β-actin triggers accumulation of H3K27ac in B to A switching regions. a) β-actin KO over WT Log2FC in rlog/VST normalized H3K27ac counts for each 50kb bin in switching and stable compartments. FIG S5. [file 13059_2023_2853_MOESM1_ESM.pdf]
