# Supplementary material for: β-actin mediated H3K27ac changes demonstrate the link between compartment switching and enhancer-dependent transcriptional regulation
Source: Genome Biol. 2023 Jan 25;24:18. doi: 10.1186/s13059-023-02853-9 (PMC9875490; doi:10.1186/s13059-023-02853-9)
Supplement: Supplementary file 3 — Additional file 3. [file 13059_2023_2853_MOESM3_ESM.docx]

**1^st^ round**

**Reviewer 1**

By reanalyzing published datasets, this manuscript showed that compartmental switching, upon reprogramming and β-actin loss, is associated with changes in enhancer activity, measured by chromatin accessibility and H3K27ac. This finding is potentially interesting, but many conclusions are premature. Furthermore, most points in this manuscript are not novel and the causal relationship proposed by the tittle was not well-supported by their data.

Major

1. In the tittle, the authors claimed that "Compartment-switching drives transcriptional change by regulating the activity of distal regulatory elements." This indicates causal relationship among compartment switching, distal elements and transcription. However, data presented in this manuscript only suggest association. Appreciated rescue and time-point experiments are needed to draw such a strong conclusion.

2. Abstract: "…compartment reorganization are equally important for enhancer-dependent transcriptional regulation." Which kind of quantification indicate the "equally importance"?

3. Fig 1d,f: How did the chromatin accessibility of a gene be measured? ATAC-seq mainly detects highly accessible regions, such as promoters and enhancers. As the lengths of genes are highly variable, it is hard to measure the "chromatin accessibility of a gene" accurately. A more appropriate strategy is to look at the ATAC-seq signal around TSS, say 2 Kb.

4. Generally, H3K4me1 is epigenetic markers for enhancer, and H3K27ac is epigenetic markers for both active enhancer and promoter. That means, intergenic H3K27ac may not sufficient to define enhancer, and they may also stand for potential promoters for novel transcripts.

5. "…transcriptional changes observed upon compartment switching were dependent on changes in gene accessibility." This conclusion is premature without examining the ATAC-seq data around TSS. This reviewer suggests to plot heatmaps centered by TSSs with ATAC-seq and H3K27ac data.

6. "accessibility of intergenic regions appeared more closely linked to transcriptional change than gene accessibility." No data support this point.

7. Fig 1g. The data is impressive. What is the genomic distribution of these upregulated H3K27ac peaks? Are they distal or near TSS? Can similar patterns be seen after MEFs reprogramed into iPSCs?

8. Fig 1h was not clear. What did the red and blue boxes indicate? If red and blue genes were compared, were they statistically significant?

9. Fig 2c. Why enhancer numbers increase in all categories of NED upon actin loss? For MEF vs ESC, loss of enhancers were seen in both DE and NDE. How can one explain the data?

10. Would author observe some key genes or key pathway members involving in switched compartment? Any key regulator (e.g., transcription regulators) play roles in regulating these gene and/or pathways?

Minor

1. In abstract, information regarding results is too short to demonstrate their significance and impact.

2. It looks like authors using |log2FC| >0.5 as cutoff to screen significant cases (figure 1d), this reviewer felt that the cutoff is too loose. If results altered once using |FC| >2 (|log2FC|>1)?

3. "…irrespective of whether they overlapped a gene or not (Fig. S2a)." Fig S2a was mis-cited in this paragraph, should be Fig. S3a.

**Reviewer 2**

The manuscript by Mahmood et al. presents an analysis of how 3D genome organization, namely A/B compartment switching, relates to transcriptional changes and chromatin accessibility, using Hi-C, RNA-seq, ATAC-seq, and ChIP-seq datasets in MEF cells undergoing reprogramming or subjected to loss of nuclear beta-actin. A/B compartmentalization represents a key feature of genome architecture, and switching compartments has been shown to associate with a change in a gene's transcriptional activity. Here, the authors carry out a detailed analysis of this association, with additional data on chromatin states, and conclude that switching-associated transcriptional changes rely in large part on changing the activity of enhancers. Thus, similar to other published reports, this is another integration of Hi-C, chromatin, and expression changes to understand their complex relationship, yet this study does appear to provide several unique insights.

Initially, the authors show that as previously proposed, compartment switching, which in their case occurs during MEF reprogramming or depletion of beta-actin, correlates to large scale changes in expression, such that A to B transitions correlate to genes going down and B to A transition to genes going up. Interestingly, such switching-associated differentially expressed genes (DEGs) do not exhibit consistent changes in chromatin accessibility (ATAC-seq), yet overall, chromatin accessibility changes correlate to compartment switching. The authors find that this discrepancy is explained by such accessibility changes occurring frequently at intergenic regions, as opposed to promoters and genes. They provide evidence that such intergenic regions within switching compartments are in fact enhancers, although this evidence is somewhat indirect. The evidence is based mainly on enhancer identification, using ABC model, which integrates H3K27acetylation and ATAC-seq reads as "Activity" and Hi-C-derived contact frequency as "Contact" measures. The authors also find that the Activity component is more able to predict enhancers than the Contact component, suggesting that chromatin marks may define enhancers more than long-range contacts. An interesting side observation in the manuscript is that depletion of beta-actin leads to increased H3K27 acetylation, although the authors do not delve into this deeper. Overall, the authors' main conclusion that it is the chromatin state of enhancers that drives transcriptional changes during compartment switching is interesting and would be of broad interest, but additional analysis would clarify and support this idea further:

1. There is somewhat of a disconnect between the information presented in Figure 1D, which demonstrate that DEGS in A to B and B to A switching do not correlate well to ATAC-seq, and the ABD enhancers shown in Figure 3C-D. Can the authors identify enhancers for genes/DEGS in 1D that do not show ATAC-seq changes (or for all of these genes, but show the ATAC-unchanged genes separately) and look at whether these enhancers show ATAC-seq changes? Although ATAC data may be part of the ABC identification, perhaps the authors can just use the H3K27acetyl signal or use a low threshold for ATAC-seq data. It seems important for the authors' main claim that they demonstrate that the genes that switch compartments and expression are accounted for by enhancers that change accessibility.

2. In Figure 2C, the NDE genes also show significant changes in enhancers upon compartment switching, but it is stated that they show no changes (pg. 7, lines 44-47) - can the authors address this? It is not totally unexpected that genes that do not change expression and switch compartments also show a change in their enhancers, but this should perhaps be mentioned and discussed further.

3. The analysis of Activity vs. Contacts contributions shown in Figure 3 is intriguing, but long-range contact frequencies, as identified by C techniques, generally vary less than ChIP-seq or ATAC-seq signal, and are more difficult to score for extent of change. It's difficult to tell whether this analysis has any biological meaning, given this limitation and the specific cut-offs or approaches the ABC model uses. It is quite possible that the majority of enhancer-promoter loops don't change upon compartment switching but it seems the authors should look into this point more carefully before making this claim.

4. Minor point - the sentence "Most genes located in A to B and B to A regions were significantly up and downregulated respectively" (pg. 5, lines 51-52) seems backwards.

**Authors’ response**

Reviewer #1: By reanalyzing published datasets, this manuscript showed that compartmental switching, upon reprogramming and β-actin loss, is associated with changes in enhancer activity, measured by chromatin accessibility and H3K27ac. This finding is potentially interesting, but many conclusions are premature. Furthermore, most points in this manuscript are not novel and the causal relationship proposed by the tittle was not well-supported by their data.

Authors response: We thank the reviewer for the constructive feedback. Based on the reviewer’s comments we have now performed extensive additional analysis including both sequencing experiments and computational analysis. We have made substantial revisions to the text and believe that the new data has considerably improved our manuscript which highlights several novel findings. These include:

oThe ability of β-actin levels to trigger and rescue changes in H3K27ac

oThe ability of β-actin levels to trigger and rescue changes in enhancer activity and compartment organization

oThe correlation between accessibility changes at non-promoter / intergenic regions and transcriptional changes within switching compartments

oThe impact of compartment switching on gain or loss of enhancers

oThe greater relative contribution of Activity (accessibility and acetylation) changes compared to Contact (Interaction frequency) changes in enhancer annotation using the ABC model

Major

1. In the tittle, the authors claimed that "Compartment-switching drives transcriptional change by regulating the activity of distal regulatory elements." This indicates causal relationship among compartment switching, distal elements and transcription. However, data presented in this manuscript only suggest association. Appreciated rescue and time-point experiments are needed to draw such a strong conclusion.

Authors response: To address the reviewer’s concerns, we have now performed rescue experiments using previously generated cell lines (1) expressing NLS-tagged β- actin in the knockout background. We have performed RNA-Seq, ATAC-Seq and H3K27ac ChIP-Seq experiments in these cells to demonstrate the impact of β-actin dependent changes in acetylation on enhancer regulation and transcription within compartment switching regions. Our data shows that reintroduction of β-actin into the knockout background, rescues B to A compartment switching (Fig. S4a) and reverses the associated increase in H3K27ac (Fig. 2a and Fig. S4b) and transcription (Fig. 2c) within these regions. Furthermore, reintroduction of β-actin also reduces the average number of enhancers associated with B to A switching genes (Fig.3c i & ii) resulting in decreased gene expression (Fig. 4 i & ii). We believe our data now strongly hints at a causal relationship between compartment-switching, transcriptional change, and enhancer activity. Nevertheless, to further address the reviewer’s concern, we have also revised the manuscript title to focus more specifically on the novel role of actin mediated acetylation changes in regulating distal regulatory elements and compartment switching. The new title now reads as follows: β-actin mediated H3K27ac changes demonstrate the link between compartment switching and enhancer-dependent transcriptional change

2. Abstract: "...compartment reorganization are equally important for enhancer- dependent transcriptional regulation." Which kind of quantification indicate the "equally importance"?

Authors response: We thank the reviewer for pointing this out. We did not intend to imply equal importance in the mathematical sense. Our aim was only to highlight the fact that both compartment organization and enhancer activity are influenced by epigenetic changes. Based on the reviewer’s feedback, we have replaced the word “equally” with “also” and the sentence now reads as follows: “Our findings demonstrate that epigenetic changes which drive compartment reorganization are also important for enhancer-dependent transcriptional regulation”.

3. Fig 1d,f: How did the chromatin accessibility of a gene be measured? ATAC-seq mainly detects highly accessible regions, such as promoters and enhancers. As the lengths of genes are highly variable, it is hard to measure the "chromatin accessibility of a gene" accurately. A more appropriate strategy is to look at the ATAC-seq signal around TSS, say 2 Kb.

Authors response: We agree with the reviewer that it is not trivial to accurately measure the “chromatin accessibility of a gene” using ATAC-Seq. As proposed by the reviewer, we had initially used TSS accessibility to study the relationship between accessibility and transcription. A potential drawback of this approach, however, is the fact that it is limited to known TSSs and ignores unannotated/novel promoters. As described in the manuscript, we therefore used the average accessibility of ATAC-Seq peaks overlapping a gene (and its 750bp upstream promoter region) as a proxy for “gene accessibility”. The advantage of this approach is that it incorporates changes in accessibility of all highly accessible regions (ATAC-Seq peaks) along the length of the gene whether they are annotated as promoters or not. Similarly, variable gene lengths do not impact this analysis because we are comparing the accessibility of the same gene in two different conditions.

However, as per the reviewer’s suggestion, we have also repeated the analysis from Figure 1d and 1f using TSS accessibility (Fig. R1). The results remain consistent with our previous analysis. In fact, even fewer genes showed changes in promoter accessibility (defined as accessibility of 1.5kb region centered on TSS) upon compartment switching compared to the previously computed gene accessibility (average of all differential ATAC peaks overlapping a gene and promoter region 750bp upstream of its TSS). This observation further highlights the importance of distal elements in regulating transcription upon compartment reorganization.

4. Generally, H3K4me1 is epigenetic markers for enhancer, and H3K27ac is epigenetic markers for both active enhancer and promoter. That means, intergenic H3K27ac may not sufficient to define enhancer, and they may also stand for potential promoters for novel transcripts.

Authors response: The precise definition of an enhancer remains a matter of active debate. In this manuscript, we have used the operational definition of an enhancer proposed by the Activity by Contact model (2). This model defines a region as an enhancer using a combination of its accessibility, acetylation and HiC contact frequency with potential promoters. We have therefore not relied solely on H3K27ac to define enhancers. While it is true that regions rich in intergenic H3K27ac may stand as promoters for novel transcripts, that does not exclude them from being potential enhancers as a promoter for one gene may act as an enhancer for another gene (3, 4). Furthermore, intergenic regions rich in H3K27ac that are annotated as enhancers by the ABC model also have a significant number of HiC contacts with potential promoters making it likely that they are also acting as enhancers.

5. "...transcriptional changes observed upon compartment switching were dependent on changes in gene accessibility." This conclusion is premature without examining the ATAC-seq data around TSS. This reviewer suggests to plot heatmaps centered by TSSs with ATAC-seq and H3K27ac data.

Authors response: As previously described, we have used change in average accessibility of ATAC-Seq peaks overlapping a gene (and 750bp upstream promoter region) as a proxy for gene accessibility. TSS associated peaks are a subset of this data and hence changes in TSS accessibility are already accounted for in our analysis. However, to address the reviewer’s concern we are also providing below heatmaps of ATAC-Seq and H3K27ac centered around TSSs (Fig. R2). The results are largely consistent with our previous analysis as only a subset of TSSs show changes in accessibility upon compartment switching. This effect is particularly pronounced for TSSs switching from B to A compartment upon actin loss. While TSSs switching from B to A compartment upon MEF to ESC reprogramming do show more noticeable changes in TSS accessibility, it should be noted that heatmaps rely on visual analysis of ATAC-Seq signal and not all changes observed in heatmaps are statistically significant.

Interestingly, changes in H3K27ac at TSSs appear more intriguing than changes in accessibility. Actin loss seems to induce accumulation of H3K27ac at TSSs in all compartments hinting at a role for actin in regulating the activity of acetyltransferases. Changes in H3K27ac upon MEF to ESC reprogramming on the other hand are largely limited to compartment switching TSSs. Surprisingly, the gain or loss of H3K27ac at TSSs does not appear to correspond with changes in accessibility in both datasets. For example, H3K27ac shows dramatic accumulation in the A compartment of β-actin knockout cells without any significant change in chromatin accessibility. These findings are consistent with a recent study showing that loss of H3K27ac at enhancers has a limited impact on chromatin accessibility and is not capable of functionally determining enhancer activity on its own (5).

6. "accessibility of intergenic regions appeared more closely linked to transcriptional change than gene accessibility." No data support this point.

Authors response: Based on the reviewer’s feedback, we have removed this sentence from the manuscript. This sentence was based on the observation that while transcriptional and accessibility changes were overrepresented in switching compartments (Fig. 1c and Fig. S3b), a large proportion of these accessibility changes were in intergenic regions (Fig. S3c). However, we agree with the reviewer that this evidence was based on correlation and have therefore omitted this sentence from the manuscript.

7. Fig 1g. The data is impressive. What is the genomic distribution of these upregulated H3K27ac peaks? Are they distal or near TSS? Can similar patterns be seen after MEFs reprogramed into iPSCs?

Authors response: We have now repeated the H3K27ac differential analysis for the ESCvsMEF dataset and included it in Figure 2. The results show that both actin depletion (Fig. 2a i) and MEF to ESC reprogramming (Fig. 2a iii) induce a noticeable increase in acetylation with the majority of H3K27ac peaks being upregulated. Furthermore, analysis of the genomic distribution of these H3K27ac peaks reveals that they are mainly present in genic or intergenic regions rather than at promoters (Fig. 2b). This effect is particularly striking in MEF to ESC reprogramming where 95% of the upregulated peaks are in genic or intergenic regions and only 5% are present at promoters.

8. Fig 1h was not clear. What did the red and blue boxes indicate? If red and blue genes were compared, were they statistically significant?

Authors response: Since Figure 1h was somewhat confusing, we have replaced it with Fig. 2C to increase clarity. This figure integrates H3K27ac ChIP-Seq with RNA-Seq data for both datasets and highlights the link between acetylation and transcription. To generate this figure, we first annotated differentially expressed genes (padj<=0.05 and no logFC cuttoff) with H3K27ac peaks. Genic peaks were annotated with genes they physically overlapped while intergenic peaks were annotated to their closest gene. Based on this annotation, genes were classified into four groups: i) genes associated with one or more upregulated H3K27ac peaks, ii) genes associated with one or more downregulated H3K27ac peaks iii) genes associated with both upregulated and down regulated H3K27ac peaks iv) genes associated with unchanging H3K27ac peaks. The result of this analysis clearly showed that genes associated with upregulated H3K27ac peaks exhibited increased average expression in both datasets.

9. Fig 2c. Why enhancer numbers increase in all categories of NED upon actin loss? For MEF vs ESC, loss of enhancers were seen in both DE and NDE. How can one explain the data?

Authors response: The boxplots in Fig. 3C (previously Fig. 2C) show that the median change in the number of enhancers for NDE genes upon actin loss is zero for all categories. As pointed out by the reviewer, it is interesting that the interquartile range for all categories is above zero but that does not mean that all NDE genes show an increase in enhancers upon actin loss (as shown by the whiskers of the boxplot and outliers plotted below zero). While we can only speculate as to why NDE genes in the actin dataset show a slightly higher average increase in enhancers, as discussed in the manuscript, one possibility might be that β-actin loss affects acetyltransferase activity and hence influences enhancer function on a global level. This is consistent with the genome-wide increase in H3K27ac observed upon β-actin loss (Fig. 2a i)

10. Would author observe some key genes or key pathway members involving in switched compartment? Any key regulator (e.g., transcription regulators) play roles in regulating these gene and/or pathways?

Authors response: In both datasets, up to 8% of all switching genes were involved in transcription regulator activity (Fig. R3). In the actin dataset switching genes included developmental regulators such as Bone Morphogenetic Proteins family of growth factors Bmp3 and Bmp6, Fibroblast growth factor Fgf5, and Sox21: a key transcription factor regulating differentiation. In the ESCvsMEF dataset, switching compartments contained more than 30 zinc finger transcription factors and more than 20 homeobox protein involved in development. Examples include Tbx and Nkx genes (involved in differentiation and myocardial developments), Pax7 (involved in skeletal muscle differentiation), and Otx1 (involved in brain development) among others.

Minor

1. In abstract, information regarding results is too short to demonstrate their significance and impact.

Authors response: We appreciate the reviewer’s feedback. As we had submitted this manuscript as a short communication, we were constrained by the recommended word limit for the abstract. We have now revised the abstract to better highlight the significant and impact of our results.

2. It looks like authors using |log2FC| >0.5 as cutoff to screen significant cases (figure 1d), this reviewer felt that the cutoff is too loose. If results altered once using |FC| >2 (|log2FC|>1)?

Authors response: We agree with the reviewer that the cutoff used to screen accessibility changes is not very strict. However, we have deliberately chosen a liberal cutoff to define accessibility changes. This approach emphasizes the fact that majority of the compartment switching genes don’t show changes in average ATAC signal even when using a very liberal cutoff to classify as gene as differentially accessible. In fact, our analysis shows that the results are not very sensitive to the cutoff used for values up to|log2FC|>1. These results indicate that genes classified as not changing accessibility simply don’t have any overlapping differential ATAC peaks and those that overlap differential ATAC peaks are classified correctly by our approach even using low cutoffs. To highlight this effect, we have used a cutoff of |log2FC|>0 & |log2FC|<0 to define accessibility changes in Figure 1D. A sensitivity analysis using different cutoffs is provided below in Fig. R4.

3. "...irrespective of whether they overlapped a gene or not (Fig. S2a)." Fig S2a was mis-cited in this paragraph, should be Fig. S3a.

Authors response: We thank the reviewer for pointing this out and have revised the manuscript accordingly.

Reviewer #2: The manuscript by Mahmood et al. presents an analysis of how 3D genome organization, namely A/B compartment switching, relates to transcriptional changes and chromatin accessibility, using Hi-C, RNA-seq, ATAC-seq, and ChIP-seq datasets in MEF cells undergoing reprogramming or subjected to loss of nuclear beta- actin. A/B compartmentalization represents a key feature of genome architecture, and switching compartments has been shown to associate with a change in a gene's transcriptional activity. Here, the authors carry out a detailed analysis of this association, with additional data on chromatin states, and conclude that switching- associated transcriptional changes rely in large part on changing the activity of enhancers. Thus, similar to other published reports, this is another integration of Hi-C, chromatin, and expression changes to understand their complex relationship, yet this study does appear to provide several unique insights.

Initially, the authors show that as previously proposed, compartment switching, which in their case occurs during MEF reprogramming or depletion of beta-actin, correlates to large scale changes in expression, such that A to B transitions correlate to genes going down and B to A transition to genes going up. Interestingly, such switching-associated differentially expressed genes (DEGs) do not exhibit consistent changes in chromatin accessibility (ATAC-seq), yet overall, chromatin accessibility changes correlate to compartment switching. The authors find that this discrepancy is explained by such accessibility changes occurring frequently at intergenic regions, as opposed to promoters and genes. They provide evidence that such intergenic regions within switching compartments are in fact enhancers, although this evidence is somewhat indirect. The evidence is based mainly on enhancer identification, using ABC model, which integrates H3K27acetylation and ATAC-seq reads as "Activity" and Hi-C-derived contact frequency as "Contact" measures. The authors also find that the Activity component is more able to predict enhancers than the Contact component, suggesting that chromatin marks may define enhancers more than long- range contacts. An interesting side observation in the manuscript is that depletion of beta-actin leads to increased H3K27 acetylation, although the authors do not delve into this deeper. Overall, the authors' main conclusion that it is the chromatin state of enhancers that drives transcriptional changes during compartment switching is interesting and would be of broad interest, but additional analysis would clarify and support this idea further:

Authors response: We thank the reviewer for the constructive feedback. We have now included additional experiments and analyses to further support our findings. As pointed out by the reviewer, we have now also increased emphasis on the novel finding that β-actin levels can have a significant impact on H3K27ac and included it in the manuscript title.

1. There is somewhat of a disconnect between the information presented in Figure 1D, which demonstrate that DEGS in A to B and B to A switching do not correlate well to ATAC-seq, and the ABD enhancers shown in Figure 3C-D. Can the authors identify enhancers for genes/DEGS in 1D that do not show ATAC-seq changes (or for all of these genes, but show the ATAC-unchanged genes separately) and look at whether these enhancers show ATAC-seq changes? Although ATAC data may be part of the ABC identification, perhaps the authors can just use the H3K27acetyl signal or use a low threshold for ATAC-seq data. It seems important for the authors' main claim that they demonstrate that the genes that switch compartments and expression are accounted for by enhancers that change accessibility.

Authors response: As per the reviewer’s suggestion, we have generated heatmaps showing change in ATAC and H3K27ac signal at the ABC-enhancers of A to B and B to A switching genes for both datasets (Fig. R3). As requested, we have divided these heatmaps into enhancers of genes that change accessibility and genes that don’t (Fig. R5). Heatmaps based on the ESCvsMEF dataset confirm that enhancers associated with non-differentially accessible B to A switching genes show significant increase in both accessibility and acetylation while enhancers of non-differentially accessible A to B switching genes show the opposite epigenetic changes. In contrast, changes in the accessibility and acetylation of promoters of switching genes are much less pronounced (Fig. R2).

While we have also repeated this analysis for the actin dataset, the results are less striking than the ESCvsMEF dataset possibly due to the smaller number of switching genes and therefore a smaller sample size. Furthermore, loss of β-actin seems to result in global dysregulation of H3K27ac and hence both promoters and enhancers in all compartments show increased acetylation making it hard to distinguish between enhancer and promoter-based regulation.

2. In Figure 2C, the NDE genes also show significant changes in enhancers upon compartment switching, but it is stated that they show no changes (pg. 7, lines 44-47) - can the authors address this? It is not totally unexpected that genes that do not change expression and switch compartments also show a change in their enhancers, but this should perhaps be mentioned and discussed further.

Authors response: We agree with the reviewer that NDE genes also show some gain or loss of enhancers. We did not intend to imply that no NDE genes gained or lost enhancers and the manuscript only mentioned that NDE genes do not show a change in average enhancers per gene. We have now further revised the referenced text to emphasize this point and have also included a brief discussion on the possible reasons for this observation. The text describing Fig 3C (previously Fig 2C) now reads as follows:

“On the other hand, NDE genes located in the same switching compartments also gained or lost some enhancers but showed negligible change in the median number of enhancers per gene. While these results highlight the general link between enhancer activity and transcription, they also show that gain or loss of enhancers alone is not sufficient to influence gene expression in the absence of downstream factors such as transcription factor availability and other forms of transcriptional/post-transcriptional gene regulation. This observation is consistent with the concept of activatable and occluded genes which respectively require either chromatin independent mechanisms like transcription factors or chromatin based derepression mechanisms in addition to enhancer activity to be properly activated (26).”

3. The analysis of Activity vs. Contacts contributions shown in Figure 3 is intriguing, but long-range contact frequencies, as identified by C techniques, generally vary less than ChIP-seq or ATAC-seq signal, and are more difficult to score for extent of change. It's difficult to tell whether this analysis has any biological meaning, given this limitation and the specific cut-offs or approaches the ABC model uses. It is quite possible that the majority of enhancer-promoter loops don't change upon compartment switching but it seems the authors should look into this point more carefully before making this claim.

Authors response: We agree with the reviewer’s point about the technical limitations of C-based techniques and that it is not possible to definitively comment on the relative contribution of long-range interactions using just HiC data. However, we believe that our finding that ABC enhancer annotations are much more heavily influenced by epigenetic state than contact frequency are still important for future studies utilizing the ABC model for identifying enhancers. Whether this observation also reflects the underlying biology of enhancer function or not, however, is a topic for future investigations and would require additional experiments using higher resolution data possibly from techniques such as Capture-C or Micro-C. We have now revised the text to emphasize the importance of our results in the context of the ABC-model and mentioned that the biological significance of this analysis is an area of future research. The following text has been added in the discussion of the results of Fig. 3:

“However, due to technical limitations of detecting low frequency chromatin interactions using HiC, the nature of these findings remains preliminary. Future studies utilizing higher resolution contact maps, and cutting-edge techniques such as Capture-C (30) and Micro-C (31) would be essential for probing the interaction landscape of specific enhancers and definitively determining the relative contribution of epigenetic state and interaction frequency in regulating enhancer function.”

4. Minor point - the sentence "Most genes located in A to B and B to A regions were significantly up and downregulated respectively" (pg. 5, lines 51-52) seems backwards.

Authors response: We thank the reviewer for pointing this out and have revised the manuscript accordingly.

REFERENCES

1. S. R. Mahmood, X. Xie, N. Hosny El Said, T. Venit, K. C. Gunsalus, P. Percipalle, β-

actin dependent chromatin remodeling mediates compartment level changes in 3D

genome architecture. Nat. Commun. 12, 5240 (2021).

2. C. P. Fulco, J. Nasser, T. R. Jones, G. Munson, D. T. Bergman, V. Subramanian, S.

R. Grossman, R. Anyoha, B. R. Doughty, T. A. Patwardhan, T. H. Nguyen, M. Kane, E.

M. Perez, N. C. Durand, C. A. Lareau, E. K. Stamenova, E. L. Aiden, E. S. Lander, J.

M. Engreitz, Activity-by-contact model of enhancer–promoter regulation from thousands of CRISPR perturbations. Nat. Genet. 51, 1664–1669 (2019).

3. M. S. Kowalczyk, J. R. Hughes, D. Garrick, M. D. Lynch, J. A. Sharpe, J. A. Sloane-

Stanley, S. J. McGowan, M. De Gobbi, M. Hosseini, D. Vernimmen, J. M. Brown, N. E.

Gray, L. Collavin, R. J. Gibbons, J. Flint, S. Taylor, V. J. Buckle, T. A. Milne, W. G.

Wood, D. R. Higgs, Intragenic enhancers act as alternative promoters. Mol. Cell. 45,

447–458 (2012).

4. O. Mikhaylichenko, V. Bondarenko, D. Harnett, I. E. Schor, M. Males, R. R. Viales,

E. E. M. Furlong, The degree of enhancer or promoter activity is reflected by the levels

and directionality of eRNA transcription. Genes Dev. (2018),

doi:10.1101/gad.308619.117.

5. T. Zhang, Z. Zhang, Q. Dong, J. Xiong, B. Zhu, Histone H3K27 acetylation is

dispensable for enhancer activity in mouse embryonic stem cells. Genome Biol. 21, 45

(2020).

**2^nd^ round**

**Reviewer 1**

My concerns were well addressed, and the manuscript is now much improved.

**Reviewer 2**

The authors have addressed my concerns and have provided the requested extra analysis, explanation, and re-writing of key points.
